# Improving Seed Morphology and Germination Potential in Australian Native Grasses Using Seed Enhancement Technologies

**DOI:** 10.3390/plants12132432

**Published:** 2023-06-23

**Authors:** Bianca Berto, Todd E. Erickson, Alison L. Ritchie

**Affiliations:** 1 School of Biological Sciences, The University of Western Australia, Crawley, WA 6009, Australia; alison.ritchie@uwa.edu.au; 2 Kings Park Science, Department of Biodiversity, Conservation and Attractions, Kings Park, WA 6005, Australia; todd.erickson@uwa.edu.au; 3 Centre for Engineering Innovation: Agriculture and Ecological Restoration, School of Agriculture and Environment, The University of Western Australia, Crawley, WA 6009, Australia

**Keywords:** acid digestion, direct seeding, flash flaming, hydropriming, restoration technologies, seed-based restoration, seed handling

## Abstract

Difficult to handle seed material and poor germination commonly limit the uptake of native grasses in restoration and commercial-scale seeding efforts. Seed enhancement technologies (SETs) offer valuable solutions for improving the handling of seed material and optimising germination. This study considered eight widespread Australian native grasses; two representative of Mediterranean to temperate climates (‘cool-climate’ species) and six representative of arid to subtropical climates (‘warm-climate’ species). Through a series of experiments, this study logically selected and applied SET treatments to improve seed handling and germination for each study species. Seed handling was prioritised and addressed using flash flaming and/or acid digestion, while hydropriming was used following seed-handling treatments to enhance germination. Flash flaming and acid digestion were both applied to successfully reduce or remove bulky floret structures while maintaining or improving germination. Flaming at 110 ± 10 °C with continuous exposure for 10 min and acid digestion concentrations of 75–80% with exposure times of 1–2.5 min were generally successful. Sub-optimal concentrations of sulphuric acid often compromised germination. Hydropriming did not improve germination outcomes when applied following flaming or acid digestion. Optimising SETs for germination, emergence and establishment in different environments, and the viability and costs of application on larger seed batches are key considerations for the implementation and upscaling of SETs in the future.

## 1. Introduction

Difficult to handle seed material is a prolific challenge among native grasses and is one of the greatest limitations preventing their uptake in restoration and commercial-scale seeding efforts [1,2]. The floret surrounding an individual seed, comprised of the palea and lemma, can have complex morphologies and appendages including prominent hairs, lobes, and awns [3,4]. These structures cause grass florets to become entangled in cleaning (i.e., dehulling) or direct seeding equipment, limiting or reducing the ability of this material to be passed efficiently when required [1,2,5,6,7,8]. In addition to these seed-handling challenges, physiological dormancy is common throughout the Poaceae family [9,10]. In grasses, physiological processes within the seed and/or mechanical restrictions created by floret structures can contribute to dormancy maintenance, causing low or asynchronous germination [10,11,12].

Seed enhancement technologies (SETs) can play a valuable role in ensuring effective use of native seed and encompass a range of post-harvest treatments applied to seeds to enhance seed handling and delivery, germination, plant performance, and/or the tolerance of seeds and plants to environmental stress [8,13,14,15,16,17]. While some SETs are widely used in crop species and the commercial seed industry, others are unique to native seed use [7,14,18]. Examples include flash flaming and acid digestion (more specialised to native species) [19,20] and seed priming (widely used in agriculture and native species) [13,21].

Flash flaming is a technique which allows seed material to be rapidly and repeatedly passed through a flame to gradually singe off unwanted structures (e.g., fine hairs on grass florets), in turn improving seed handling and increasing bulk density [5,20,22]. Acid digestion can achieve similar outcomes by exposing seed material to sulphuric acid, which dissolves or ‘digests’ both fine and thickened appendages [1,19]. Each of these treatments have been associated with improving germination by providing a physiological germination cue and/or reducing the mechanical restrictions imposed by floret structures [1,6,19,20,23].

Seed priming is a common method of widening the environmental conditions for which non-dormant seeds germinate and often assists in overcoming low, slow, or asynchronous germination [21]. Priming in water (i.e., hydropriming) or in osmotically controlled solutions (i.e., osmopriming) involves the imbibition and redrying of seeds to commence, but not complete, the germination process [13,21]. Additives, such as the smoke-derived germination stimulant karrikinolide, can be included in the priming solution to deliver chemicals to the seed which target certain physiological processes [6,8]. Priming can also increase tolerance to drought and salinity and/or enhance seedling growth [17,24,25,26,27].

SET selection can be guided by understanding the germination biology and which barriers (handling, germination, environmental) present the greatest challenge to direct-seeding success [18]. For instance, if a species has slow, asynchronous germination, priming may be an appropriate SET. However, applying an SET which only improves seed germination may be redundant if seed handling is a substantial issue (or vice versa) [28]. Where multiple barriers to plant establishment exist (e.g., seed handling and germination), using a combination of SETs (e.g., flaming and priming) may be beneficial [6].

The effect of SETs is often species specific, and variations in the method of SET application can dramatically influence recruitment responses [1,5,6,13]. For instance, mixed germination responses under laboratory conditions have been observed for flash flaming [1,5,20,29]. Many parameters of the flaming process can be adjusted including exposure duration, flame size, and flame temperature [5]. Ineffective application (e.g., excessive duration and flame intensity) has been shown to decrease germination [1,5,22], while optimized application (determined through testing germination responses to different application methods) can maintain or improve germination [5,6,22]. Similar instances have been observed with acid digestion [1,19] and seed priming [6,17,26]. Therefore, testing and applying SETs to produce the best germination, emergence, and establishment responses is required to advance our understanding and adoption of SETs in large-scale restoration and commercial contexts.

The aim of this study was to (1) understand the germination biology of the study species, and (2) use this information to logically select and apply SETs for eight Australian native grasses typical of temperate to Mediterranean climates (two species) and arid to subtropical climates (six species). The selected species have various seed-handling and establishment challenges and are of value for both ecological restoration and commercial farming at large scales (e.g., pasture and rangeland species), making them ideal candidates for SET application.

## 2. Results

### 2.1. Germination Biology (Experiment 1)

#### Temperature Regimes

Maximum germination (MG, as a percent) was largely unaffected by the different temperature regimes tested in each species, though florets tested on KAR_1_ agar and clean seeds tended to have higher MG under cooler temperature regimes (Table 1, Table 2 and Table 3; Supplementary Material I, Table S1). For instance, MG was significantly higher under cooler temperature regimes (winter > spring > summer) in *N. alopecuroidea* when cleaned to seeds (4–22% higher; *p* < 0.01; Table 1). Likewise, *C. ambiguus*, *E. obtusa*, and *E. aurea* had higher MG under the autumn compared to the summer temperature regime when cleaned to seeds (25%, 11%, and 22% higher, respectively; *p* < 0.001; Table 2). Intact florets of *E. obtusa* also had higher MG under autumn temperatures (KAR_1_ agar only, 15% higher; *p* < 0.001; Table 2).

Time to 50% of maximum germination (T50m, in d) was generally shorter under warmer temperature regimes. *Neurachne alopecuroidea* had shorter T50m under spring and summer temperatures compared to winter (by up to 3.37 d; *p* < 0.001; Table 1), though *R. caespitosum* had shorter T50m under winter and spring temperatures compared to summer (by up to 3.73 d; *p* < 0.05; Table 1). In the warm-climate species, T50m was shorter under summer temperatures compared to autumn for *A. inaequiglumis* florets (by 1.61 d; *p* < 0.001), *C. ambiguus* florets (water agar only, by 0.85 d; *p* < 0.001), *C. obtectus* florets (by 0.27–0.37 d; *p* < 0.05), and *E. obtusa* florets and seeds (KAR_1_-agar only, by 0.34–0.92 d; *p* < 0.05) (Table 2). However, germination rate (GR, as number of seeds germinating per d (g/d)) was faster under autumn temperatures in *C. ambiguus* florets (by 1.86–2.37 g/d; *p* < 0.001) and *E. obtusa* florets tested on KAR_1_ (by 1.33 g/d *p* < 0.01).

### 2.2. Removal of Floret Structures

Removal of floret structures (i.e., cleaning to seeds) affected MG in all species except *R. caespitosum* (Table 1, Table 2 and Table 3; Table S2). MG was lower for clean seeds compared to florets by 19–36% in *N. alopecuroidea*, 24–57% in *C. ambiguus*, and 15–41% in *E. aurea* (*p* < 0.05). Contrastingly, MG increased by 53–60% in *C. fallax* (*p* < 0.001), 5–9% in *C. obtectus* (*p* < 0.05), and 21–31% in *E. obtusa* (*p* < 0.01) in clean seeds compared to florets. T50m was generally shorter for all study species (except *R. caespitosum*) following removal of the floret structures (*p* < 0.05). GR was influenced by cleaning to seed only in *E. obtusa* when tested on KAR_1_ under autumn temperatures (faster by 1.38 g/d; *p* < 0.05).

### 2.3. KAR_1_

Responses to KAR_1_ were inconsistent and infrequent across the study species (Table 1, Table 2 and Table 3; Table S2). Clean seeds of *N. alopecuroidea* had lower MG when exposed to KAR_1_ under the spring temperature regime only (*p* < 0.001; Table 1). Of the warm-climate species, MG was negatively affected by KAR_1_ for seeds of *C. ambiguus* under autumn temperatures only (29% lower; *p* < 0.001), and positively affected by KAR_1_ in florets of *E. obtusa* (9–23% higher; *p* < 0.05) (Table 2).

**Table 1:** Maximum germination (MG), time to 50% germination (T50m), and germination rate (GR), (parameters *d*, *e*, and *b* of the *drc* package, respectively) for the cool-climate species when florets and clean seeds were tested under each temperature regime and on water agar or KAR_1_ agar. Values listed are mean ± standard error.

Species	Temp Regime	Treatment	Maximum Germination (*d*)	T50m (*e*)	Germination Rate (*b*)
***Neurachne alopecuroidea***	Winter (18/7 °C)	Floret	87 ± 4.25	11.08 ± 0.32	3.79 ± 0.7
		Floret + KAR_1_	84 ± 3.17	11.09 ± 0.26	5.14 ± 0.98
		Seed	79 ± 2.31	5.92 ± 0.24	3.72 ± 0.83
		Seed + KAR_1_	72 ± 1.93	6.54 ± 0.19	5.22 ± 1.06
	Spring (26/13 °C)	Floret	87 ± 2.84	7.71 ± 0.24	3.46 ± 0.54
		Floret + KAR_1_	92 ± 2.34	8.12 ± 0.21	4.77 ± 0.88
		Seed	68 ± 1.89	4.69 ± 0.21	3.58 ± 0.65
		Seed + KAR_1_	56 ± 1.82	4.92 ± 0.27	3.94 ± 0.89
	Summer (33/18 °C)	Floret	82 ± 2.85	7.87 ± 0.26	3.53 ± 0.6
		Floret + KAR_1_	85 ± 2.63	7.6 ± 0.24	3.66 ± 0.57
		Seed	57 ± 1.71	4.46 ± 0.21	4.47 ± 1.1
		Seed + KAR_1_	61 ± 1.76	4.44 ± 0.2	4.10 ± 0.93
***Rytidosperma caespitosum***	Winter (18/7 °C)	Floret	35 ± 2.29	7.62 ± 0.5	3.35 ± 1.12
		Floret + KAR_1_	33 ± 2.48	7.99 ± 0.55	3.17 ± 1.04
		Seed	32 ± 1.9	6.58 ± 0.47	3.53 ± 1.21
		Seed + KAR_1_	31 ± 1.79	5.72 ± 0.47	3.22 ± 1.1
	Spring (26/13 °C)	Floret	30 ± 3.33	6.54 ± 0.68	2.31 ± 0.96
		Floret + KAR_1_	36 ± 2.12	7.01 ± 0.46	3.23 ± 0.94
		Seed	27 ± 6.17	8.6 ± 1.54	1.78 ± 0.92
		Seed + KAR_1_	22 ± 4.21	9.27 ± 1.21	2.53 ± 1.48
	Summer (33/18 °C)	Floret	26 ± 5.1	10.27 ± 1.23	2.52 ± 1.36
		Floret + KAR_1_	32 ± 4.09	8.62 ± 0.82	2.34 ± 0.9
		Seed	23 ± 5.19	8.04 ± 1.44	1.9 ± 1.11
		Seed + KAR_1_	35 ± 5.49	8.18 ± 1.02	1.87 ± 0.71

**Table 2:** Maximum germination (MG), time to 50% germination (T50m), and germination rate (GR), (parameters *d*, *e*, and *b* of the *drc* package, respectively) for the warm-climate species when florets and clean seeds were tested under each temperature regime and on water agar or KAR_1_ agar. Values listed are mean ± standard error.

Species	Temp Regime	Treatment	Maximum Germination (*d*)	T50m (*e*)	Germination Rate (*b*)
***Aristida inaequiglumis***	Autumn (32/17 °C)	Floret	91 ± 2.39	4.49 ± 0.18	4.14 ± 1.37
		Floret + KAR_1_	94 ± 2.33	4.67 ± 0.13	2.81 ± 0.54
		Seed	-	-	-
		Seed + KAR1_1_	-	-	-
	Summer (39/25 °C)	Floret	91 ± 2.58	2.88 ± 0.14	2.05 ± 0.23
		Floret + KAR1_1_	91 ± 2.18	3.22 ± 0.20	2.63 ± 0.38
		Seed	-	-	-
		Seed + KAR1_1_	-	-	-
***Chrysopogon fallax***	Autumn (32/17 °C)	Floret	34 ± 3.38	3.42 ± 0.94	2.79 ± 2.13
		Floret + KAR1_1_	32 ± 4.10	3.73 ± 4.28	4.27 ± 17.66
		Seed	87 ± 2.92	1.83 ± 0.11	2.79 ± 1.17
		Seed + KAR1_1_	87 ± 2.61	1.92 ± 0.08	3.91 ± 2.74
	Summer (39/25 °C)	Floret	36 ± 3.80	3.22 ± 0.62	2.17 ± 0.97
		Floret + KAR1_1_	30 ± 2.99	2.81 ± 0.60	2.86 ± 1.50
		Seed	96 ± 13.10	0.70 ± 0.40	0.81 ± 0.63
		Seed + KAR1_1_	85 ± 3.73	1.40 ± 0.27	1.97 ± 1.02
***Cymbopogon ambiguus***	Autumn (32/17 °C)	Floret	96 ± 2.29	4.56 ± 0.14	3.94 ± 0.53
		Floret + KAR1_1_	100 ± 2.25	4.30 ± 0.11	4.40 ± 0.77
		Seed	72 ± 2.10	2.67 ± 0.16	2.84 ± 0.49
		Seed + KAR1_1_	43 ± 2.28	2.65 ± 0.26	2.75 ± 0.91
	Summer (39/25 °C)	Floret	94 ± 3.14	3.71 ± 0.19	2.08 ± 0.32
		Floret + KAR1_1_	95 ± 3.38	3.97 ± 0.20	2.03 ± 0.33
		Seed	47 ± 2.09	2.67 ± 0.25	2.80 ± 0.71
		Seed + KAR1_1_	49 ± 1.95	2.26 ± 0.16	3.27 ± 1.04
***Cymbopogon obtectus***	Autumn (32/17 °C)	Floret	92 ± 1.58	3.92 ± 0.07	4.72 ± 1.01
		Floret + KAR1_1_	95 ± 1.57	3.93 ± 0.07	5.05 ± 1.20
		Seed	97 ± 2.21	0.82 ± 0.31	1.59 ± 0.68
		Seed + KAR1_1_	99 ± 1.43	1.20 ± 0.32	2.89 ± 1.51
	Summer (39/25 °C)	Floret	90 ± 1.62	3.55 ± 0.16	4.51 ± 1.47
		Floret + KAR1_1_	93 ± 1.69	3.66 ± 0.11	3.97 ± 0.90
		Seed	99 ± 1.49	0.96 ± 0.71	2.83 ± 2.89
		Seed + KAR1_1_	100 ± 3.14	0.32 ± 0.59	1.23 ± 1.30
***Eriachne obtusa***	Autumn (32/17 °C)	Floret	54 ± 1.95	4.04 ± 0.17	3.94 ± 1.22
		Floret + KAR1_1_	77 ± 2.08	3.28 ± 0.55	4.19 ± 0.14
		Seed	85 ± 1.63	5.53 ± 4.85	3.12 ± 0.68
		Seed + KAR1_1_	84 ± 1.62	4.82 ± 3.04	2.94 ± 0.57
	Summer (39/25 °C)	Floret	53 ± 2.41	2.56 ± 0.82	3.64 ± 0.28
		Floret + KAR1_1_	62 ± 2.88	1.95 ± 0.39	3.85 ± 0.26
		Seed	74 ± 1.63	3.29 ± 0.58	2.25 ± 0.09
		Seed + KAR1_1_	73 ± 1.67	3.33 ± 0.87	2.02 ± 0.07
***Eulalia aurea***	Autumn (32/17 °C)	Floret	91 ± 3.52	3.14 ± 1.48	3.42 ± 0.58
		Floret + KAR1_1_	93 ± 2.95	6.76 ± 17.76	3.99 ± 2.34
		Seed	76 ± 3.56	2.33 ± 0.79	2.06 ± 0.15
		Seed + KAR1_1_	65 ± 8.40	2.48 ± 0.73	2.14 ± 0.14
	Summer (39/25 °C)	Floret	95 ± 3.43	2.46 ± 0.40	2.91 ± 0.22
		Floret + KAR1_1_	95 ± 3.10	2.99 ± 0.62	3.06 ± 0.30
		Seed	54 ± 4.06	1.96 ± 0.98	1.93 ± 0.24
		Seed + KAR1_1_	65 ± 8.40	1.16 ± 0.79	1.37 ± 0.35

### 2.4. Seed Enhancement Technologies (Experiments 2 and 3)

#### 2.4.1. Flaming

**Figure 1:**
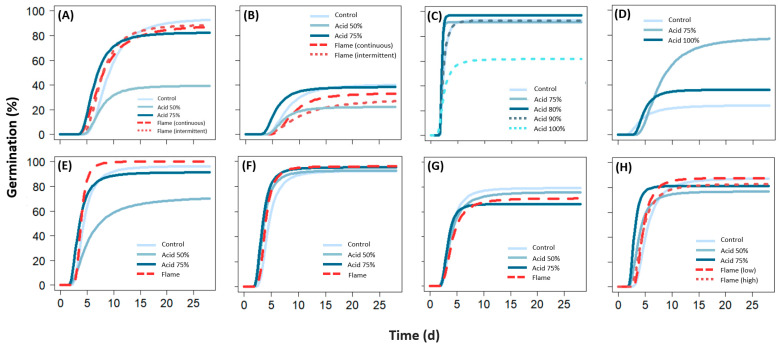
Germination curves (as per three-parameter Weibull model) for (**A**) *Neurachne alopecuroidea*, (**B**) *Rytidosperma caespitosum*, (**C**) *Aristida inaequiglumis*, (**D**) *Chrysopogon fallax*, (**E**) *Cymbopogon ambiguus*, (**F**) *Cymbopogon obtectus*, (**G**) *Eriachne obtusa*, and (**H**) *Eulalia aurea* following flaming and acid digestion treatments. The different concentrations of acid digestion used for each species, and the flaming variations ‘continuous’ and ‘intermittent’ for the cool climate species, and ‘low’ or ‘high’ for *E. aurea* (corresponding to flaming temperatures of 110 ± 10 °C and 150 ± 10 °C, respectively), are listed. Pairwise comparisons of the different flaming and acid digestion application methods and parameter estimates for each species are available in Supplementary Material II, Tables S3–S5. A breakdown of the cost of application for flash flaming and acid digestion can be found in Supplementary Material III, Table S6.

**Figure 2:**
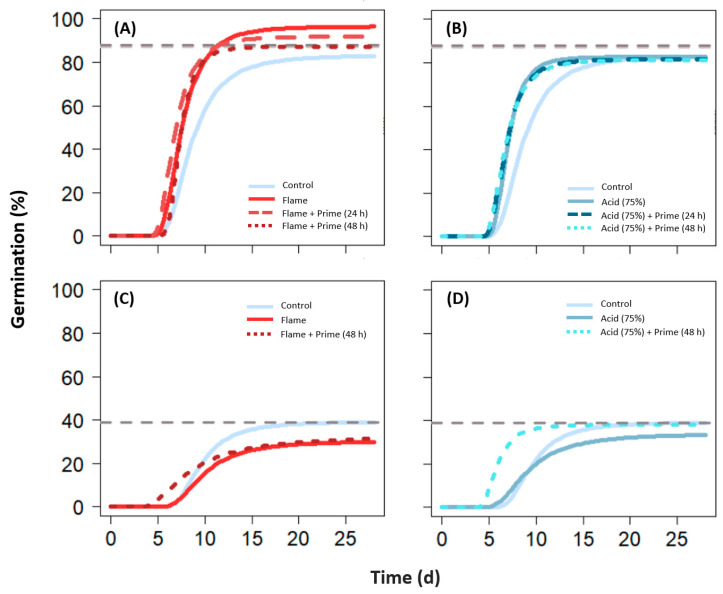
Germination curves (as per three-parameter Weibull model) for (**A**,**B**) *Neurachne alopecuroidea* and (**C**,**D**) *Rytidosperma caespitosum* following treatment with (**A**,**C**) flaming and (**B**,**D**) acid digestion treatments in combination with hydropriming. Grey dashed lines represent maximum germination achieved from hydropriming alone. Pairwise comparisons of the different SET treatments and parameter estimates for each species are available in Tables S4 and S5.

MG was unaffected by flaming in the cool-climate species in Experiment 2 (Figure 1, Table 3; Supplementary Material II, Table S3), though subsequent testing in Experiment 3 resulted in lower MG in *R. caespitosum* (by 9%; *p* < 0.05; Table S4), and higher MG for *N. alopecuroidea* compared to untreated florets (by 14%; *p* < 0.001) (Figure 2). Of the warm-climate species, MG was unaffected in all species except *E. obtusa* where germination was lower for flamed florets (by 8%; *p* < 0.01) (Figure 1, Table 3). T50m was shorter for *N. alopecuroidea* (by 0.87–1.36 d in both experiments; *p* < 0.001), *C. obtectus* (by 0.4 d; *p* < 0.05), and *E. aurea* (by 0.53–0.73 d; *p* < 0.01) for flamed compared to untreated florets (Figure 1 and Figure 2, Table 3; Table S3).

#### 2.4.2. Acid Digestion

Compared to untreated (control) florets, acid digestion reduced MG in for both concentrations tested in *N. alopecuroidea* (by 11–54%; *p* < 0.001), and the 50% concentration in *R. caespitosum* (19% lower; *p* < 0.001) (Figure 1; Table S3). When retested in Experiment 3, the 75% concentration resulted in similar MG outcomes to the control in both of the cool-climate species (Figure 2; Table S4). In the warm-climate species, acid digestion produced similar MG outcomes to the control in *A. inaequiglumis* (75% and 90% concentration), *C. ambiguus* (75% conc.), *C. obtectus* (both conc.), and *E. obtusa* (50% conc.) (Figure 1, Table 3; Table S3). MG was higher when compared to the control in *A. inaequiglumis* using an 80% concentration (by 4%; *p* < 0.05), and *C. fallax* for both concentrations (by 12–55%; *p* < 0.001) (Figure 1). Using a 100% concentration solution in *A. inaequiglumis* reduced germination by 31% compared to the control (*p* < 0.001; Figure 1).

T50m was shorter for *N. alopecuroidea* (by 1.22–2.21 d; *p* < 0.05; for both conc.), *R. caespitosum* (by 1.83 d; *p* < 0.05; 75% conc., Experiment 3 only), *C. ambiguus* (by 0.82 d; *p* < 0.001; 75% conc.), *C. obtectus* (by 0.82–0.95 d; *p* < 0.001; for both conc.), and *E. aurea* (by 1.34–1.97 d; *p* < 0.001; for both conc.) compared to the control following acid digestion (Figure 1 and Figure 2, Table 3; Table S3). GR was faster only in *E. aurea* for 75% concentration compared to the control (by 1.48 g/d; *p* < 0.05; Table S3).

#### 2.4.3. Hydropriming

Hydropriming (Experiment 3, cool-climate species only) when used alone or in combination with flaming or acid digestion had no effect on MG compared to the control (Figure 2, Table 3; Table S4), except for the flaming and hydropriming (24 h) combination in *N. alopecuroidea* (9% higher than control; *p* < 0.001). Compared to hydropriming alone, flaming (continuous) with hydropriming (24 h) resulted in higher MG (by 5%; *p* < 0.05), and acid digestion with priming combinations resulted in lower MG (by 5–7%; *p* < 0.05) for *N. alopecuroidea* (Table S4).

Hydropriming combinations with flaming and acid digestion had a shorter T50m than the control (by 0.8–1.56 d; *p* < 0.001), flaming alone (by 0.59 d for 48 h hydropriming comparisons only; *p* < 0.05), and hydropriming alone (by 0.59–1.35 d; *p* < 0.001) for *N. alopecuroidea* (Table 3; Table S4). This was accompanied by a faster GR for flaming used in combination with hydropriming (48 h) treatments in *N. alopecuroidea* compared to the control, flaming alone, and 48 h hydropriming alone (by 2.16–3.17 g/d; *p* < 0.05). In *R. caespitosum*, T50m was also shorter for hydropriming when used alone (by 1.35 d; *p* < 0.05) and in combination with acid digestion (by 3.19 d; *p* < 0.05) compared to the control (Table 3; Table S4).

**Table 3:** Summary of the key results for the treatments tested in each experiment.

**Experiment 1: Germination Biology**	
Treatment	Key findings
Temperature regimes	The majority of species demonstrated the capacity to germinate equally well under different temperature regimes. Cooler temperatures were favoured when exposed to KAR_1_ and/or cleaned to seed. T50m was generally shorter under warmer temperatures.
Removing floret structures	Decreased tolerance to higher temperatures (e.g., *N. alopecuroidea, C. ambiguus, E. obtusa,* and *E. aurea*). Alleviated seed dormancy (e.g., *C. fallax* and *E. obtusa*). Generally reduced T50m.
KAR_1_	Neutral to inconsistent responses to exposure.
**Experiments 2 and 3: SET Application**	
Treatment	Key findings
Flash flaming	Fine hairs associated with floret successfully reduced with neutral effects on germination under the settings used (110 ± 10 °C). Including cooling periods (intermittent flaming) had no effect on germination. T50m often shorter (e.g., *N. alopecuroidea*, *C. obtectus*, *E. aurea*).
Acid digestion	Concentrations of 75–80% with exposure times of 1–2.5 min were generally effective for appendage reduction while maintaining (or enhancing) germination capacity. Using 50% concentration was less effective for appendage reduction and detrimental to germination in some species (e.g., *N. alopecuroidea*, *C. ambiguus*).
Hydropriming	Neutral effects on maximum germination when used alone, mixed effects when used in combination with other SETs. Overall faster germination.

## 3. Discussion

Seed enhancement technologies provide valuable solutions to improving restoration outcomes and the commercial success of native species, particularly those with high forage value [1,5,6,13,14,17,23,30,31]. This study demonstrates the importance of selecting and testing SETs which address seed-handling challenges while considering germination biology. The germination biology of each study species highlighted key processes which may be limiting (e.g., complex dormancy mechanisms) or of value to the uptake of native grasses in restoration and commercially (e.g., consistently high germination over a range of conditions). Flash flaming and acid digestion were successfully applied to reduce bulky appendages associated with poor seed handling while maintaining or improving germination outcomes, though inappropriate application methods commonly resulted in germination losses. Acid digestion was also able to overcome mechanical restrictions to germination that had previously been identified. Where hydropriming was explored, it was unable to overcome physiological barriers causing low or slow germination. Linking SET responses to germination biology is a valuable tool for understanding which barriers certain SETs are best suited to overcoming.

### 3.1. Understanding Germination Biology

Seed dormancy prevents germination at times when seasonal conditions are conducive to germination, but seedling survival is unlikely [11,32]. Once non-dormant (>75% germination [31]), the expression of germination is typically over a wider environmental envelope. Of the eight study species explored, physiological dormancy was observed in *R. caespitosum*, *C. fallax*, and *E. obtusa* (<75% germination for intact florets on water agar). All other species achieved relatively high maximum germination (>75%) when intact florets were tested on water agar across all temperature regimes, suggesting an absence of dormancy, or that dormancy was alleviated prior to experimental use (e.g., via seed ageing during storage).

When moisture is not limiting, germination is typically highest at the temperatures which coincide with the normal recruitment/rainfall season [32,33]. The majority of the species in this study, however, demonstrated the capacity to germinate equally well under temperature regimes outside of those associated with their known preferred recruitment season. For instance, florets of the cool-climate species germinated equally well across the winter, spring, and summer regimes, despite their recruitment events being associated with winter and spring [34,35]. Likewise, florets of the warm-climate species had similar germination under both the summer and autumn temperature regime, despite summer being the known recruitment season [33] (with the exception of *E. obtusa* which showed a preference for autumn temperatures). The overall high levels of germination observed across the contrasting temperature regimes may suggest some level of germination plasticity once in non-dormant state. This could be as a result (or cause) of the wide geographical distributions associated with each of the study species [35]. However, the temperatures explored in this study may not have approached the minimum and maximum germination temperature thresholds for these species, thereby not yielding a significant response. For instance, germination declines have been observed for warm-climate *Triodia* species when maximum temperature exceeded 35 °C [36].

In half of the study species, the process of removing floret structures decreased their tolerance to higher temperatures. This trend was observed for *N. alopecuroidea*, *C. ambiguus*, *E. obtusa,* and *E. aurea*. The role of floret structures surrounding the seed in providing protection against sub-optimal temperatures (among other adverse environmental conditions) is well documented [37]. In the Poaceae family, the floret structures surrounding the seed are most commonly associated with dispersal functions, though these structures may also protect the seed during germination and establishment [4]. For instance, the floret structures for the species studied here may have provided beneficial insulation for seeds during germination at sub-optimal temperatures.

For some species in this study, however, removing floret structures aided in alleviating seed dormancy. Germination of *C. fallax* and *E. obtusa* increased by up to 60% and 31%, respectively, following the removal of the floret structures. For each of these species, the mechanical restrictions to embryo growth imposed by the floret structures are a key mechanism contributing to physiological dormancy. This process is common in studies of the Poaceae family which remove or weaken floret structures either manually, or via treatments such as acid digestion and flash flaming [1,12,23,38].

Germinating florets containing seeds or extracted seeds in the presence of KAR_1_, a smoke-derived germination stimulant, was also used to determine the overall germination potential of each species and treatment. Where beneficial, KAR_1_ could then be used as an additive in SET applications (e.g., in hydropriming treatments). Overall, responses to KAR_1_ exposure were inconsistent across species, temperature regimes, and seed units tested (florets or cleaned seed). Several other studies of Poaceae have also reported a lack of response or mixed responses to fire-related treatments (e.g., smoke, KAR_1_) [9,39]. The lack of KAR_1_ response in this study was likely linked to the non-dormant nature of the seed batches (i.e., aged seeds are potentially less responsive to KAR_1_ exposure [12]). Future applications of KAR_1_ use in these species should consider evaluations on freshly collected material.

### 3.2. Seed Enhancement Application

#### 3.2.1. Flash Flaming

Mixed responses to flaming have been observed across several species, with flaming settings (in particular torch/flame size and exposure duration) being known to influence germination [1,5,20]. The flaming temperatures and exposure durations used in this study (110 ± 10 °C applied continuously for 10 min) were relatively low compared to those used in other flaming experiments. For example, Pedrini et al. [1] applied flaming for up to 60 min, and Berto et al. [6] used flaming temperatures of 160 °C for up to 20 min. The settings used in this study elicited few and small negative responses, and may therefore suit multiple species and seed batches. The flaming variations of ‘intermittent’ and ‘continuous’ flaming of cool-climate species, and increasing flaming temperatures to 150 ± 10 °C for *E. aurea*, had no significant effect on germination responses.

This study aimed to select flaming settings reflecting temperatures which may be experienced in the soil seedbank during a natural fire event. The temperatures experienced by a seed during wildfires and the exposure duration can vary considerably depending on the fuel source, conditions, and location of the seed in the soil profile [40,41,42]. Temperatures of 100–600 °C have been recorded at the soil surface for grass fires, with these rapidly peaking and dropping within a few minutes [41,43]. However, below soil surface temperatures of 50–150 °C can be maintained for up to 60 min at a depth of 2 cm [43].

Additionally, seeds of different species have different lethal temperature thresholds [42]. Ruckman et al. [44] found several native rangeland grasses were tolerant of temperatures of up to 250 °C for 4 min, while other studies have found temperatures of 50–110 °C for up to 2 min can have positive, neutral, or negative effects on germination in Mediterranean grasses [45,46]. An improved understanding of lethal temperature thresholds in seeds of various species and exploring a greater range of flaming temperatures and durations would be highly applicable for flash-flaming protocol development.

#### 3.2.2. Acid Digestion

Acid digestion produced contrasting germination responses, which were largely driven by variations in the treatment application (i.e., different concentrations and exposure durations). Neutral to positive germination responses were achieved for all species (except *N. alopecuroidea*) when acid digestion was applied at the most suitable concentration and duration, with these treatments also tending to reduce T50m. Mixed responses to acid digestion have been observed across and within studies, with the concentration and exposure duration known to influence germination outcomes [1,19,47]. However, the diversity of treatment application and purpose across studies makes it challenging to generalise which concentrations or durations may be broadly successful.

While some studies aim to reduce or remove bulky floret structures [1,19], others aim to alleviate dormancy via scarification [47,48,49,50]. Treatment applications can range in concentration of 25–100% and exposure durations of 1–100 min depending on the treatment objective [1,39,47,48,49,50]. Concentrations of 25–75% are commonly harmless to germination outcomes [19,47], though many studies have applied 95–100% sulphuric acid for long durations (>10 min) with germination benefits recorded [49,51].

In this study, the 75% concentration tended to be most effective for appendage removal while maintaining (or enhancing) germination. Optimal acid digestion treatments on average had an exposure duration of 1–2.5 min, regardless of the concentration. Whether this is a true trend highlighting a window of optimal exposure duration, or an artefact of similar concentrations (75–80%), producing the best germination responses remains unclear. The strength of this finding is also limited by different sulphuric acid concentrations being applied to each species for varied durations, and it is therefore not possible to determine whether germination responses were driven by concentration, exposure duration, or an interaction between these.

Future studies linking seed germination biology and particular anatomical and morphological traits with responses to acid digestion would be valuable for guiding appropriate application methods. For example, higher concentrations and longer exposure durations may be suitable in species with thickened structures surrounding the seed and/or a deep level of dormancy, while lower concentrations may be better suited to species with fine structures surrounding the seed and low levels of dormancy (as was observed in this study).

#### 3.2.3. Hydropriming

Hydropriming following flaming and acid treatments was used for the cool-climate species (*N. alopecuroidea* and *R. caespitosum*) to improve maximum germination and germination speed and synchronicity (e.g., T50m). Hydropriming has been widely used across the agricultural industry to enhance germination performance and establishment success [21], and there are a growing number of examples of hydropriming benefiting germination in native seeds [6,17,27,52]. The hydropriming treatments tested in this study, however, had neutral to negative effects in *N. alopecuroidea* and neutral effects in *R. caespitosum.*

In *N. alopecuroidea*, hydropriming tended to reduce the positive effects of flaming, and had no influence on the effects of acid digestion. Florets of *N. alopecuroidea* which were hydroprimed only (i.e., not pre-treated with flaming or acid digestion) also showed neutral germination responses. While hydropriming had neutral effects in *R. caespitosum*, the treatment was able to restore germination following flaming as previously observed in Berto et al. [6]. The contrasting responses to hydropriming may be due to the uncontrolled nature of imbibition which can risk seed damage in some species [17,21]. Although previous studies have found benefits to using hydropriming following the application of other SETs [6], it is possible that applying certain SETs prior to hydropriming may exacerbate the risk of uncontrolled and damaging imbibition. In these instances, osmopriming may offer a suitable alternative to hydropriming as the osmotic potential of the priming solution can be controlled, thereby minimising risk of damage to the seed [21]. The link between seed priming following pre-treatments such as flaming or acid digestion warrants further investigation.

### 3.3. Scaled Application and Future Research of SETs

If SETs are to be adopted at scale in restoration and commercial industries, both the success of the treatment in overcoming a particular barrier to plant establishment in the targeted environment as well as the viability and cost of application on larger batches must be considered [14,53]. Flash flaming and acid digestion were both suitable for removing bulky seed appendages associated with seed-handling issues. Often it was clear as to which of these two SETs was most effective for each study species due to the suitability of the treatment to reduce unwanted appendages, or because one treatment produced better germination outcomes than the other. However, in instances where several SETs produce similar improvements in overcoming plant establishment barriers, other considerations such as logistics, scalability, and/or environmental factors should be prioritised.

The flaming technology is currently more time, resource, and cost effective than acid digestion, having been up-scaled to treat large volumes of seed (up to 3 L) in short periods of time (e.g., 10 min treatment duration) [5,29]. By contrast, acid digestion requires an equal volume of sulphuric acid solution to the volume of seed being treated, and treating large volumes is currently a lengthily and logistically complicated process as this technology has not yet been up-scaled [8]. Furthermore, the estimated costs of flash flaming and acid digestion from this study demonstrated that flaming is two to five times more cost-effective than acid digestion (based on the methodology used in this study). Therefore, flash flaming is currently the preferred SET for large-scale implementation and commercial uptake.

Further to these logistic considerations is the need to better understand the long-term effects of SETs on plant establishment. While this study considers the effects of SETs on germination within a laboratory setting, it is important to understand the effects on subsequent life stages such as emergence and early establishment over a range of environmental conditions. For instance, priming is well known to enhance tolerance of seeds and seedlings to environmental stressors [8], though whether treatments such as acid digestion and flash flaming have effects on germination and/or establishment under more heterogeneous field conditions is unknown.

While it is important to undergo the iterative process of SET testing under laboratory conditions, it is not necessarily conclusive. To further understand how SETs perform under contrasting environmental conditions, testing SETs over a range of soil types and rainfall scenarios in glasshouse and field studies would provide valuable insight into the viability of applying these SETs at scale. Alternately, germination and emergence testing over temperature and moisture gradients under laboratory conditions could highlight the unique sets of environmental conditions and scenarios where SETs may have the greatest benefit and applicability.

## 4. Conclusions

The value of native grasses in ecosystem function and commercial forage systems is widely acknowledged, though they remain underutilised and underrepresented in restoration and commercial industries due to seed-handling and germination challenges. Improving the ability to disperse and establish native grass seeds over large scales via the use of SETs is a critical step toward the adoption of native grasses. Flash flaming, acid digestion, and seed priming all provide useful solutions to overcoming the barriers to native grass seed use. To ensure successful application of these technologies, logical selection and optimised application must be implemented, the performance of SETs under contrasting environments requires evaluation, and the viability and cost of application on large seed quantities must be considered.

## 5. Materials and Methods

### 5.1. Study Species

**Figure 3:**
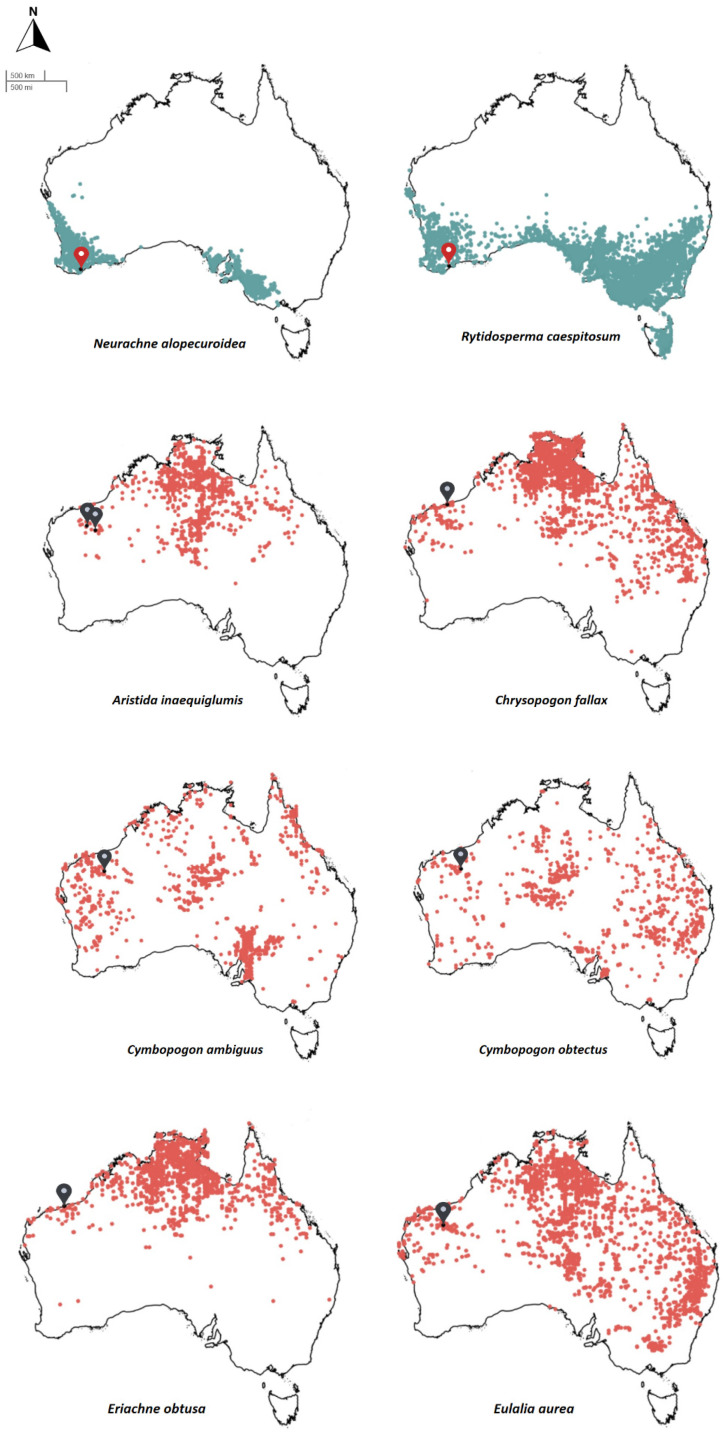
Species distribution maps for the cool-climate (teal) and warm-climate (red) species. All seed collections are from Western Australia, with collection locations for each study species indicated by a red or black pin for the cool- and warm-climate species, respectively. Maps were generated from Atlas of Living Australia using species occurrence records data. Further seed collection information is provided in Table S7.

**Figure 4:**
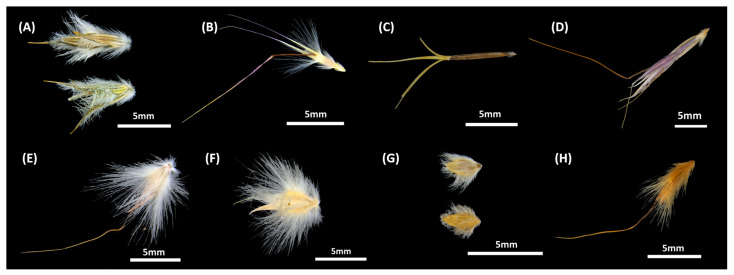
High resolution images of untreated florets of each of the study species demonstrating the hairs, awns, and appendages associated with the floret structures. Species are labelled as (**A**) *Neurachne alopecuroidea*, (**B**) *Rytidosperma caespitosum*, (**C**) *Aristida inaequiglumis*, (**D**) *Chrysopogon fallax*, (**E**) *Cymbopogon ambiguus*, (**F**) *Cymbopogon obtectus*, (**G**) *Eriachne obtusa*, and (**H**) *Eulalia aurea*.

Eight widely distributed native perennial grasses were selected for this study, two from temperate to Mediterranean (‘cool’) climates and six from arid to subtropical (‘warm’) climates. The cool-climate species included *Neurachne alopecuroidea* R.Br. and *Rytidosperma caespitosum* (Gaudich.) Connor & Edgar, while the warm-climate species included *Aristida inaequiglumis* Domin, *Chrysopogon fallax* S.T.Blake, *Cymbopogon ambiguus* A.Camus, *Cymbopogon obtectus* S.T.Blake, *Eriachne obtusa* R.Br., and *Eulalia aurea* (Bory) Kunth. Note, however, that some of the species are extant across both climatic regions (Figure 3). The cool-climate species occur in climatic regions where mean annual precipitation (MAP) and temperature (MAT) are in the ranges of 150–1500 mm and 10–22 °C, respectively, while the warm-climate species occur in climatic regions where MAP and MAT are in the ranges of 125–2000 mm and 14–29 °C, respectively (Supplementary Material IV, Table S7). The major rainfall season coincides with the winter months for the cool-climate species and the summer months for the warm-climate species. Each of the study species has prominent hairs and/or awns and appendages associated with the floret structures (Figure 4).

### 5.2. Study Overview

A series of laboratory experiments were performed to test the germination biology characteristics of each species (Experiment 1), apply SETs to overcome seed-handling challenges (Experiment 2), and apply SETs to further improve germination where required (Experiment 3). To assess germination biology, germination testing over a range of seasonal temperatures with or without removing external floret structures and exposure to the smoke-derived compound karrikinolide (KAR_1_; 3-methyl-2H-furo[2,3-c]pyran-2-one (synthesized following the methods of [54]) was conducted for each species (Table 4). Florets and seeds were tested in the presence of KAR_1_ to isolate key ecological processes (i.e., fire) which may influence physiological dormancy, if present [11,31,55], and as a possible tool to aid in the selection of suitable SETs (e.g., flaming, KAR_1_ delivery via priming). Seed enhancements were selected based on the morphological characteristics and germination biology of each species and included flash flaming and acid digestion (Experiment 2), and hydropriming (Experiment 3). Flash flaming and acid digestion were selected primarily to address challenges associated with seed morphology (i.e., to reduce/remove bulky floret structures), while hydropriming was selected primarily to overcome low and/or slow germination. A range of different methods of applying each SET were tested to determine the optimal treatment method for each species (Table 5).

### 5.3. Experiment 1: Germination Biology

Florets containing seeds (hereafter ‘florets’) and cleaned seeds (i.e., floret structures, comprised of the palea and lemma, removed; hereafter ‘seeds’) of each species were tested on agar with or without the addition of KAR_1_ under different seasonal temperature regimes (Table 4). Floret structures were removed from the seeds by gently rubbing florets between ribbed rubber mats [56]. This was carried out for all species except *A. inaequiglumis* where cleaning to seed has not been possible to date for this species as the seeds are prone to breakage due to the elliptical, tightly bound floret shape [56]. Cleaned seeds were checked carefully under a microscope to ensure that the endosperm and embryo were not damaged during cleaning.

Germination tests were performed on agar prepared with reverse osmosis (RO) water (0.7% *w*/*v*) or with RO water containing a 0.67 μM concentration of KAR_1_, hereafter referred to as water agar and KAR_1_ agar, respectively. Each germination test used four 90 mm Petri dishes (replicates) containing 25 filled florets or seeds. A mixture of manually separating florets from non-target material (i.e., stalks, chaff), vacuum aspiration (‘Zig Zag’ Selecta, Machinefabriek BV, Enkhuizenm the Netherlands), and X-ray analysis (Faxitron MX-20 digital X-ray cabinet, Tucson, AZ, USA) were used to identify and remove empty florets. Prior to being transferred to Petri dishes, florets and seeds were sterilised in a 2% (*w*/*v*) calcium hypochlorite (Ca[OCl]_2_) solution for 30 min, alternating for 10 min cycles under vacuum pressure (i.e., on/off/on at −80 kPa).

Florets and seeds were germinated in incubators (Contherm Biosyn 6000CP; Contherm Scientific Ltd., Wellington, New Zealand) with a 12 h light/dark cycle at temperatures representative of typical seasonal conditions corresponding to rainfall events sufficient for germination. For the cool-climate species, these temperature regimes included 18/7 °C (winter), 26/13 °C (spring), and 33/18 °C (summer) (derived from [55]), while the temperature regimes tested for the warm-climate species included 39/25 °C (summer) and 32/17 °C (autumn) (derived from the Restoration Seed Bank (RSB) Initiative (see [57]) germination protocols). Germination tests ran for 28 d, with germination recorded every 2–3 d during peak germination to ensure data were detailed enough to allow for analysis of the germination rate, then 1–2 times per week thereafter. Seeds were considered germinated when the radicle was greater than one-third of the length of the floret [12].

### 5.4. Experiment 2: SET Applications to Improve Seed Handling

Flash flaming and acid digestion were applied to target species with seed-handling challenges. Treatment selection for each species and application methods were selected based on the published literature [5,6] and pilot studies (Supplementary Material V, Figure S1). All germination tests were conducted at the optimal temperature regime, as per the germination results from Experiment 1 (18/7 °C for *R. caespitosum*, 26/13 °C for *N. alopecuroidea*, and 32/17 °C for the warm-climate species). Floret material was prepared for germination testing following the methodology outlined in Experiment 1 and was tested on water agar only. The costs of resources to apply flash flaming and acid digestion treatments were recorded and the resultant cost of treating 1 L of seed material for each of these SETs was estimated. While not directly measured in this study, the seed handling and flowability improvements associated with techniques such as flaming have been well documented [5,20,22].

#### 5.4.1. Flash Flaming

Flash flaming was performed for all species except *A. inaequiglumis* and *C. fallax*. Flaming was conducted using the custom-built flaming machine ‘MK1’ (described in Erickson et al. [13]; Supplementary Material VI, Figure S2), using a single small flame (sensu Ling et al. [29]). Florets (1 L samples) were flamed for 10 min at 110 ± 10 °C (monitored at regular intervals using a laser thermometer; Ozito, Bangholme Australia). Deviations from this occurred for the cool-climate species (*N. alopecuroidea* and *R. caespitosum*) where ‘continuous flaming’ and ‘intermittent flaming’ were tested due to a known previous intolerance to flaming [5,6], and for *E. aurea* where a temperature of 150 ± 10 °C was maintained to more effectively remove long hairs. ‘Continuous flaming’ exposed floret material to a flame continuously for 10 min, while ‘intermittent flaming’ exposed florets to the flame for 1 min followed by a 30 s cooling period until a total flame exposure time of 10 min had been achieved (15 min total duration). Volume and weight changes were recorded for each species following flaming treatments (Table S8).

#### 5.4.2. Acid Digestion

Acid digestion was performed using various concentrations of sulphuric acid (H_2_SO_4_; reagent grade 98%; Sigma Chemicals, Willetton, Western Australia). Concentrations and exposure durations were in the ranges of 50–100% and 40 s–1 h, respectively, and were selected based on preliminary testing which targeted morphological changes to the floret structures (Table 5; Figure S1). Sulphuric acid was diluted in RO water at the appropriate volumes to achieve each concentration. For each species, small (~50 mL) samples of floret material were immersed in the appropriate sulphuric acid solution and agitated intermittently to ensure thorough exposure. The treated material was immediately neutralised in sodium hydrogen carbonate solution (8.4 g L^−1^ NaHCO_3_, Sigma-Aldrich, St. Louis, MO, USA) and rinsed thoroughly in RO water before being dried for a minimum of 48 h at 15 °C and 15% relative humidity.

### 5.5. Experiment 3: SET Applications to Provide Additional Germination Benefits

#### Priming

Hydropriming was performed for the cool-climate species only using a custom-built priming unit (Supplementary Material VII, Figure S3). Priming cylinders were filled with 1 L of RO water and were aerated (3–5 L per min) for the duration of the treatments. Priming durations of 24 and 48 h at 15 °C were selected based on previous studies [6]. Hydroprimed florets were dried for a minimum of 48 h at 15 °C and 15% relative humidity. Floret material was prepared for germination testing and tested at the optimal temperature regime as per Experiment 1 on water agar only.

### 5.6. Data Analysis

All germination data were analysed using the dose–response curve (*drc*) package in R [58,59]. Dose–response curves were fitted to the germination data over time using the 3-parameter Weibull model [60,61]. This model sets the lower limit to 0 (i.e., the lowest possible value for germination) and provides estimates for parameters *d*, *e*, and *b* which correspond to maximum germination (MG), time to 50% maximum germination (T50m), and germination rate (GR), respectively [58]. T50m provides an estimate for the number of days to reach 50% of maximum germination, while GR provides an estimate for the average number of seeds germinating per day (g/d). T50m can be significantly different as an artefact of significant differences in MG when comparing two treatments. Only in these instances is GR discussed, though GR comparisons and values for all treatments are provided in Supplementary Material I–II (Tables S1–S5).

## Figures and Tables

**Table 4:** Experiment 1 seed treatments and germination conditions tested for each species.

Species	Seed Treatment	Germination Temperatures	Growth Medium
*Neurachne alopecuroidea*	Florets Seeds	15/7 °C (winter), 26/13 °C (spring), 33/18 °C (summer)	Water agar KAR_1_ agar
*Rytidosperma caespitosum*			
*Aristida inaequiglumis*	Florets only	39/25 °C (summer), 32/17 °C (autumn)	Water agar KAR_1_ agar
*Chrysopogon fallax*	Florets Seeds		
*Cymbopogon ambiguus*			
*Cymbopogon obtectus*			
*Eriachne obtusa*			
*Eulalia aurea*			

**Table 5:** SETs tested in each study species and the details of treatment application (i.e., flaming applied continuously or intermittently, concentration and duration of acid digestion treatments, duration of hydropriming). All germination tests were performed on water agar and at the temperature regime which resulted in the best germination outcomes for each species in Experiment 1 (18/7 °C for *R. caespitosum*, 26/13 °C for *N. alopecuroidea*, and 32/17 °C for all warm-climate species).

Species	Flaming	Acid Digestion	Hydropriming	Combinations
*Neurachne alopecuroidea*	Continuous Intermittent	50% (1 h) 75% (1 min 30 s)	24 h 48 h	Flame (cont.) + Prime (24 h) Flame (cont.) + Prime (48 h) Acid (75%) + Prime (24 h) Acid (75%) + Prime (48 h)
*Rytidosperma caespitosum*	Continuous Intermittent	50% (7 min) 75% (40 s)	48 h	Flame (cont.) + Prime (48 h) Acid (75%) + Prime (48 h)
*Aristida inaequiglumis*	-	75% (6 min) 80% (2 min 30 s) 90% (1 min 45 s) 100% (1 min)	-	-
*Chrysopogon fallax*	-	75% (2 min 30 s) 100% (2 min 30 s)	-	-
*Cymbopogon ambiguus*	Continuous	50% (8 min) 75% (1 min 30 s)	-	-
*Cymbopogon obtectus*	Continuous	50% (7 min) 75% (1 min)	-	-
*Eriachne obtusa*	Continuous	50% (2 min 30 s) 75% (30 s)	-	-
*Eulalia aurea*	Continuous	50% (8 min) 75% (1 min 30 s)	-	-

## Data Availability

Not applicable.

## Supplementary Materials

The following supporting information can be downloaded at: https://www.mdpi.com/article/10.3390/plants12132432/s1, Supplementary Material I. Experiment 1 statistical analysis, Table S1. Statistical comparisons of maximum germination (d), T50m (e), and germination rate (b) between the different temperature regimes for each seed treatment (floret, floret + KAR1, seed, seed + KAR1), Table S2. Statistical comparisons of maximum germination (d), T50m (e), and germination rate (b) for each seed form (intact florets and clean seeds) on each growth medium (water-agar and KAR1-agar) within each temperature regime, Supplementary Material II. Experiment 2 and 3 statistical analysis, Table S3. Statistical comparisons of maximum germination (d), T50m (e), and germination rate (b) between the different SETs tested in Experiment 2, Table S4. Statistical comparisons of maximum germination (d), T50m (e), and germination rate (b) between the different SETs tested in Experiment 3, Table S5. Maximum germination (MG), time to 50% germination (T50m), and germination rate (GR), (parameters d, e, and b of the drc package, respectively) for all species and SETs tested in Experiments 2 and 3, Supplementary Material III. Cost of SET application, Table S6. Cost estimates of flash flaming and acid digestion application, Supplementary Material IV. Seed collection, floret fill, storage and processing, Table S7. Additional study species biology and collection information, Supplementary Material V. Selection of SETs and application methods, Figure S1. High resolution floret images before and after SET application, Supplementary Material VI. Flash flaming equipment and techniques, Figure S2. Flaming apparatus, Table S8. Volume and weight changes following flash flaming treatments, Supplementary Material VII. Priming unit, Figure S3. Priming apparatus.
