# Supplementary material for: Improving Seed Morphology and Germination Potential in Australian Native Grasses Using Seed Enhancement Technologies
Source: Plants (Basel). 2023 Jun 23;12(13):2432. doi: 10.3390/plants12132432 (PMC10346785; doi:10.3390/plants12132432)
Supplement: Supplementary file 1 [file plants-12-02432-s001.zip › plants-2433662-supplementary.pdf]

Supplementary Materials

Contents

Supplementary Material I. Experiment 1 statistical analysis 2

Table S1. Statistical comparisons of maximum germination (*d*), T50m (*e*), and germination rate (*b*) between the different temperature regimes for each seed treatment (floret, floret + KAR_1, seed, seed + KAR_1).. 2

Table S2. Statistical comparisons of maximum germination (*d*), T50m (*e*), and germination rate (*b*) for each seed form (intact florets and clean seeds) on each growth medium (water-agar and KAR_1-agar) within each temperature regime.. 4

Supplementary Material II. Experiment 2 and 3 statistical analysis 6

Table S3. Statistical comparisons of maximum germination (*d*), T50m (*e*), and germination rate (*b*) between the different SETs tested in **Experiment 2**. 6

Table S4. Statistical comparisons of maximum germination (*d*), T50m (*e*), and germination rate (*b*) between the different SETs tested in **Experiment 3**.. 8

Table S5. Maximum germination (MG), time to 50% germination (T50m), and germination rate (GR), (parameters *d*, *e*, and *b* of the *drc* package, respectively) for all species and SETs tested in Experiments 2 and 3. 10

Supplementary Material III. Cost of SET application 12

Table S6. Cost estimates of flash flaming and acid digestion application 12

Supplementary Material IV. Seed collection, floret fill, storage and processing 13

Table S7. Additional study species biology and collection information. 13

Supplementary Material V. Selection of SETs and application methods 15

Selection of SETs 15

Application method selection – flaming 15

Application method selection – acid digestion 15

Application method selection – hydropriming 16

Figure S1. High resolution floret images before and after SET application 17

Supplementary Material VI. Flash flaming equipment and techniques 19

Figure S2. Flaming apparatus 19

Table S8. Volume and weight changes following flash flaming treatments 19

Supplementary Material VII. Priming unit 20

Figure S3. Priming apparatus. 20

REFERENCES 21

Supplementary Material I. Experiment 1 statistical analysis

Table S1. Statistical comparisons of maximum germination (*d*), T50m (*e*), and germination rate (*b*) between the different temperature regimes for each seed treatment (floret, floret + KAR_1, seed, seed + KAR_1). Values indicate the value difference between the two treatments (i.e. percentage difference for maximum germination, number of days difference for T50m, and number of seeds germinating per day difference for germination rate). Where significant differences exist, this is indicated by asterisks '*', '**', and '***' indicate significance levels 0.05, 0.01, and 0.001, respectively. Positive values indicate that the treatment listed first in the comparison column had the highest value, while negative values indicate the treatment listed second in the comparison column had the highest value.

Species	Treatment	Comparison	Maximum Germination (d)	T50 (*e*)	Germination Rate (*b*)
N. alopecuroidea	Floret	winter v spring	0	3.37 ***	0.33
		winter v summer	5	3.21 ***	0.26
		spring v summer	5	-0.16	-0.07
	Floret + KAR	winter v spring	-8 *	2.97 ***	0.37
		winter v summer	-1	3.49 ***	1.48
		spring v summer	7	0.52	1.11
	Seed	winter v spring	11 **	1.23 ***	0.14
		winter v summer	22 ***	1.46 ***	-0.75
		spring v summer	11 ***	0.23	-0.89
	Seed + KAR	winter v spring	16 ***	1.62 ***	1.28
		winter v summer	11 ***	2.10 ***	1.12
		spring v summer	-5 *	0.48	-0.16
R. caespitosum	Floret	winter v spring	5	1.08	1.04
		winter v summer	9	-2.65 *	0.83
		spring v summer	4	-3.73 ***	-0.21
	Floret + KAR	winter v spring	-3	0.98	-0.06
		winter v summer	1	-0.63	0.83
		spring v summer	4	-1.61 *	0.89
	Seed	winter v spring	5	-2.02	1.75
		winter v summer	9	-1.46	1.63
		spring v summer	4	0.56	-0.12
	Seed + KAR	winter v spring	9	-3.55 ***	0.69
		winter v summer	-4	-2.46 **	1.35
		spring v summer	-13 *	1.09	0.66
A. inaequiglumis	Floret	summer v autumn	0	-1.61 ***	-2.09 *
	Floret + KAR		-3	-1.45 ***	-0.18 *
	Seed		x	x	x
	Seed + KAR		x	x	x
C. fallax	Floret		2	-0.20	-0.62
	Floret + KAR		-2	-0.92	-1.41
	Seed		9	-1.13 **	-1.98 **
	Seed + KAR		-2	-0.52	-1.94

C. ambiguus	Floret	summer v autumn	-2	-0.85 ***	-1.86 ***
	Floret + KAR		-5	-0.33	-2.37 ***
	Seed		-25 ***	0.00	-0.04
	Seed + KAR		6	-0.39	0.52
C. obtectus	Floret		-2	-0.37 *	-0.21
	Floret + KAR		-2	-0.27 *	-1.08
	Seed		2	0.14	1.24
	Seed + KAR		1	-0.88	-1.66
E. obtusa	Floret		-1	-0.40	-1.38
	Floret + KAR		-15 ***	-0.34 ***	-1.33 *
	Seed		-11 ***	-0.87	-2.24
	Seed + KAR		-11 ***	-0.92 *	-1.49
E. aurea	Floret		4	-0.51	-0.68
	Floret + KAR		2	-0.93	-3.77
	Seed		-22 ***	-0.13	-0.37
	Seed + KAR		0	-0.77 *	-1.32

Table S2. Statistical comparisons of maximum germination (*d*), T50m (*e*), and germination rate (*b*) for each seed form (intact florets and clean seeds) on each growth medium (water-agar and KAR_1-agar) within each temperature regime. Values indicate the value difference between the two treatments (i.e. percentage difference for maximum germination, number of days difference for T50m, and number of seeds germinating per day difference for germination rate). Where significant differences exist, this is indicated by asterisks '*', '**', and '***' indicate significance levels 0.05, 0.01, and 0.001, respectively. Positive values indicate that the treatment listed first in the comparison column had the highest value, while negative values indicate the treatment listed second in the comparison column had the highest value.

Species	Temp	Comparison	Maximum Germination (*d*)	T50m (*e*)	Germination Rate (*b*)
N. alopecuroidea	18/7°C (winter)	Floret v Floret + KAR	3	-0.01	-1.35
		Seed v Seed + KAR	7 *	-0.62 *	-1.50
		Floret v Seed	8	5.16 ***	0.07
		Floret + KAR v Seed + KAR	12 **	4.55 ***	-0.08
	26/13°C (spring)	Floret v Floret + KAR	-5	-0.41	-1.31
		Seed v Seed + KAR	12 ***	-0.23	-0.36
		Floret v Seed	19 ***	3.02 ***	-0.12
		Floret + KAR v Seed + KAR	36 ***	3.20 ***	0.83
	33/18°C (summer)	Floret v Floret + KAR	-3	0.27	-0.13
		Seed v Seed + KAR	-4	0.02	0.37
		Floret v Seed	25 ***	3.41 ***	-0.94
		Floret + KAR v Seed + KAR	24 ***	3.16 ***	-0.44
R. caespitosum	18/7°C (winter)	Floret v Floret + KAR	2	-0.37	0.18
		Seed v Seed + KAR	1	0.86	0.31
		Floret v Seed	3	1.04	-0.18
		Floret + KAR v Seed + KAR	2	2.27 **	-0.05
	26/13°C (spring)	Floret v Floret + KAR	-6	-0.47	-0.92
		Seed v Seed + KAR	5	-0.67	-0.75
		Floret v Seed	3	-2.06	0.53
		Floret + KAR v Seed + KAR	14	-2.26 *	0.70
	33/18°C (summer)	Floret v Floret + KAR	-6	1.65	0.18
		Seed v Seed + KAR	-12	-0.14	0.03
		Floret v Seed	3	2.23	0.62
		Floret + KAR v Seed + KAR	-3	0.44	0.47
A. inaequiglumis	39/25°C (summer)	Floret v Floret + KAR	0	-0.34	-0.58
		Seed v Seed + KAR	x	x	x
		Floret v Seed	x	x	x
		Floret + KAR v Seed + KAR	x	x	x
	32/17°C (autumn)	Floret v Floret + KAR	-3	-0.18	1.33
		Seed v Seed + KAR	x	x	x
		Floret v Seed	x	x	x
		Floret + KAR v Seed + KAR	x	x	x
C. fallax	39/25°C (summer)	Floret v Floret + KAR	6	0.41	-0.69
		Seed v Seed + KAR	11	-0.70	-1.16
		Floret v Seed	-60 ***	2.52	1.36

		Floret + KAR v Seed + KAR	-55 ***	1.41	0.89
	32/17°C (autumn)	Floret v Floret + KAR	2	-0.31	-1.48
		Seed v Seed + KAR	0	-0.09	-1.12
		Floret v Seed	-53 ***	1.59 *	0.00
		Floret + KAR v Seed + KAR	-55 ***	1.81	0.36
C. ambiguus	39/25°C (summer)	Floret v Floret + KAR	-1	-0.26	0.05
		Seed v Seed + KAR	-2	0.41	-0.47
		Floret v Seed	47 ***	1.04 **	-0.72
		Floret + KAR v Seed + KAR	46 ***	1.71 ***	-1.24
	32/17°C (autumn)	Floret v Floret + KAR	-4	0.26	-0.46
		Seed v Seed + KAR	29 ***	0.02	0.09
		Floret v Seed	24 ***	1.89 ***	1.10
		Floret + KAR v Seed + KAR	57 ***	1.65 ***	1.65
C. obtectus	39/25°C (summer)	Floret v Floret + KAR	-3	-0.11	0.54
		Seed v Seed + KAR	-1	0.64	1.60
		Floret v Seed	-9 ***	2.59 *	1.68
		Floret + KAR v Seed + KAR	-7 *	3.34	2.74
	32/17°C (autumn)	Floret v Floret + KAR	-3	-0.01	-0.33
		Seed v Seed + KAR	-2	-0.38	-1.30
		Floret v Seed	-5 *	3.10 *	3.13
		Floret + KAR v Seed + KAR	-4 *	2.73 *	2.16
E. obtusa	39/25°C (summer)	Floret v Floret + KAR	-9 *	-0.21	0.61
		Seed v Seed + KAR	1	0.23	-0.04
		Floret v Seed	-21 ***	1.39 ***	-0.73
		Floret + KAR v Seed + KAR	-11 ***	1.83 ***	-1.38 *
	32/17°C (autumn)	Floret v Floret + KAR	-23 ***	-0.15	0.66
		Seed v Seed + KAR	1	0.18	0.71
		Floret v Seed	-31 ***	0.92	-1.59
		Floret + KAR v Seed + KAR	-7 *	1.25	-1.54
E. aurea	39/25°C (summer)	Floret v Floret + KAR	0	-0.15	-0.53
		Seed v Seed + KAR	-11	0.56	0.80
		Floret v Seed	41 ***	0.98 *	0.50
		Floret + KAR v Seed + KAR	30 *	1.69 *	1.83
	32/17°C (autumn)	Floret v Floret + KAR	-2	-0.57	-3.62
		Seed v Seed + KAR	11	-0.08	-0.15
		Floret v Seed	15 *	1.36 *	0.81
		Floret + KAR v Seed + KAR	28 *	1.85	4.28

Supplementary Material II. Experiment 2 and 3 statistical analysis

Table S3. Statistical comparisons of maximum germination (*d*), T50m (*e*), and germination rate (*b*) between the different SETs tested in **Experiment 2**. Values indicate the value difference between the two treatments (i.e. percentage difference for maximum germination, number of days difference for T50m, and number of seeds germinating per day difference for germination rate). Where significant differences exist, this is indicated by asterisks '*', '**', and '***' indicate significance levels 0.05, 0.01, and 0.001, respectively. Positive values indicate that the treatment listed first in the comparison column had the highest value, while negative values indicate the treatment listed second in the comparison column had the highest value.

Species	Comparison	Maximum Germination (*d*)	T50 (*e*)	Germination Rate (*b*)
N. alopecuroidea	Control v Flame (intermittent)	5	1.07 ***	-0.22
	Control v Flame (continuous)	5	1.36 ***	0.62
	Control v Acid (50%)	54 ***	1.22 *	-0.49
	Control v Acid (75%)	11 ***	2.21 ***	-0.1
	Flame (intermittent) v Flame (continuous)	0	0.29	0.84
	Acid (50%) v Acid (75%)	-43 ***	0.99 *	0.39
R. caespitosum	Control v Flame (intermittent)	12	-1.57	0.90
	Control v Flame (continuous)	75	14.66	6.44
	Control v Acid (50%)	19 ***	0.58	-0.63
	Control v Acid (75%)	2	1.83 *	-0.20
	Flame (intermittent) v Flame (continuous)	-5	0.51	-1.32
	Acid (50%) v Acid (75%)	-17 ***	1.25	0.43
A. inaequiglumis	Control v Acid (75%)	1	0.19	-8.20
	Control v Acid (80%)	-4 *	0.19	-6.32
	Control v Acid (90%)	0	0.08	0.19
	Control v Acid (100%)	31 ***	0.09	1.70
	Acid (75%) v Acid (80%)	-5 **	0.00	1.88
	Acid (75%) v Acid (90%)	-1	-0.11	8.39
	Acid (75%) v Acid (100%)	30 ***	-0.10	9.90
	Acid (80%) v Acid (90%)	4 *	-0.11	6.51
	Acid (80%) v Acid (100%)	35 ***	-0.10	8.02
	Acid (90%) v Acid (100%)	31 ***	0.01	1.51
C. fallax	Control v Acid (75%)	-55 ***	-3.02 ***	-0.53
	Control v Acid (100%)	-12 ***	-0.76	-1.63
	Acid (75%) v Acid (100%)	43 ***	2.26 ***	-1.10
C. ambiguus	Control v Flame	-3	0.27	-1.96
	Control v Acid (50%)	25 ***	-0.49	1.32
	Control v Acid (75%)	6	0.82 ***	0.30
	Acid (50%) v Acid (75%)	-19 ***	1.31 ***	-1.02 *
	Flame v Acid (50%)	28 ***	-0.76 *	3.28 **
	Flame v Acid (75%)	9 **	0.55 *	2.26
C. obtectus	Control v Flame	-3	0.40 *	-0.75
	Control v Acid (50%)	0	0.82 ***	0.04
	Control v Acid (75%)	-2	0.95 ***	-0.15
	Acid (50%) v Acid (75%)	-2	0.13	-0.19

	Flame v Acid (50%)	3	0.42 *	0.79
	Flame v Acid (75%)	1	0.55 **	0.60
E. obtusa	Control v Flame	8 **	-0.17	0.29
	Control v Acid (50%)	3	0.15	0.63
	Control v Acid (75%)	13 ***	0.45	-0.82
	Acid (50%) v Acid (75%)	10 ***	0.3	-1.45
	Flame v Acid (50%)	-5	0.32	0.34
	Flame v Acid (75%)	5	0.62	-1.11
E. aurea	Control v Flame (110 ± 10°C)	-1	0.53 **	-0.77
	Control v Flame (150 ± 10°C)	4	0.73 ***	0.10
	Control v Acid (50%)	10 **	1.34 ***	0.06
	Control v Acid (75%)	6 *	1.97 ***	-1.48 *
	Flame (110 ± 10°C) v Flame (150 ± 10°C)	5	0.20	0.87
	Acid (50%) v Acid (75%)	-4	0.63 *	-1.54
	Flame (100-120°C) v Acid (50%)	11 ***	0.81 ***	0.83
	Flame (100-120°C) v Acid (75%)	7 **	1.44 ***	-0.71
	Flame (140-160°C) v Acid (50%)	6 *	0.61 **	-0.04
	Flame (140-160°C) v Acid (75%)	2	1.24 ***	-1.58

Table S4. Statistical comparisons of maximum germination (*d*), T50m (*e*), and germination rate (*b*) between the different SETs tested in **Experiment 3**. Values indicate the value difference between the two treatments (i.e. percentage difference for maximum germination, number of days difference for T50m, and number of seeds germinating per day difference for germination rate). Where significant differences exist, this is indicated by asterisks '*', '**', and '***' indicate significance levels 0.05, 0.01, and 0.001, respectively. Positive values indicate that the treatment listed first in the comparison column had the highest value, while negative values indicate the treatment listed second in the comparison column had the highest value.

Species	Comparison	Maximum Germination (*d*)	T50 (*e*)	Germination Rate (*b*)
N. alopecuroidea	Control v Flame (continuous)	-14 ***	0.87 ***	-0.58
	Control v Acid (75%)	0	1.24 ***	-2.11
	Flame (continuous) v Acid (75%)	14 ***	0.37	-1.53
	Control v Hydroprime (24 h)	-4	0.20	-2.28 **
	Control v Hydroprime (48 h)	-5	0.21	-1.01
	Control v Flame (continuous) + Hydroprime (24 h)	-9 ***	1.51 ***	-0.93
	Control v Flame (continuous) + Hydroprime (48 h)	-4	0.8 ***	-3.17 ***
	Control v Acid (75%) + Hydroprime (24 h)	1	1.47 ***	-1.23
	Control v Acid (75%) + Hydroprime (48 h)	2	1.56 ***	-0.89
	Hydroprime (24 h) v Hydroprime (48 h)	-1	0.01	1.27
	Flame (continuous) v Flame (continuous) + Hydroprime (24 h)	5 *	0.64 *	-0.35
	Flame (continuous) v Flame (continuous) + Hydroprime (48 h)	10 ***	-0.07	-2.59 *
	Hydroprime (24 h) v Flame (continuous) + Hydroprime (24 h)	-5 *	1.31 ***	1.35
	Hydroprime (48 h) v Flame (continuous) + Hydroprime (48 h)	1	0.59 ***	-2.16 *
	Acid (75%) v Acid (75%) + Hydroprime (24 h)	1	0.23	0.88
	Acid (75%) v Acid (75%) + Hydroprime (48 h)	2	0.32	1.22
	Hydroprime (24 h) v Acid (75%) + Hydroprime (24 h)	5 *	1.27 ***	1.05
	Hydroprime (48 h) v Acid (75%) + Hydroprime (48 h)	7 **	1.35 ***	0.12
	Flame (continuous) + Hydroprime (24 h) v Flame (continuous) + Hydroprime (48 h)	5 *	-0.71 **	-2.24
	Acid (75%) + Hydroprime (24 h) v Acid (75%) + Hydroprime (48 h)	1	0.09	0.34
	Flame (continuous) + Hydroprime (24 h) v Acid (75%) + Hydroprime (24 h)	10 ***	-0.04	-0.3
	Flame (continuous) + Hydroprime (48 h) v Acid (75%) + Hydroprime (48 h)	6 **	0.76 **	2.28
R. caespitosum	Control v Flame (continuous) 2	9 *	-0.28	0.45
	Control v Acid (75%) 2	5	0.4	1.17
	Flame (continuous) v Acid (75%)	-4	0.68	0.72
	Control v Hydroprime (48 h)	0	1.35 *	-0.13
	Control v Flame (continuous) + Hydroprime (48 h)	6	1.41	2.19

	Control v<br>Acid (75%) + Hydroprime (48 h)	1	3.19 *	-0.54
	Flame (continuous) v<br>Flame (continuous) + Hydroprime (48 h)	-3	1.69	1.74
	Hydroprime (48 h) v<br>Flame (continuous) + Hydroprime (48 h)	6	0.06	2.32
	Acid (75%) v<br>Acid (75%) + Hydroprime (48 h)	-4	2.79	-1.71
	Hydroprime (48 h) v<br>Acid (75%) + Hydroprime (48 h)	1	1.84	-0.41
	Flame (continuous) + Hydroprime (48 h) v<br>Acid (75%) + Hydroprime (48 h)	-5	1.78	-2.73

Table S5. Maximum germination (MG), time to 50% germination (T50m), and germination rate (GR), (parameters *d*, *e*, and *b* of the *drc* package, respectively) for all species and SETs tested in Experiments 2 and 3.

Species	Ex.	Treatment	Maximum germination (*d*)	SE	Germination Rate (*b*)	SE	T50 (*e*)	SE
Neurachne alopecuroidea	2	Control	94	2.46	3.34	0.39	7.98	0.18
		Flame (intermittent)	89	2.06	3.56	0.46	6.91	0.18
		Flame (continuous)	89	2.47	2.72	0.32	6.62	0.20
		Acid (50%)	40	1.89	3.83	1.25	6.76	0.43
		Acid (75%)	83	1.77	3.44	0.41	5.77	0.16
	3	Control	83	1.81	4.09	0.49	7.77	0.17
		Flame (continuous)	97	1.63	4.67	0.63	6.9	0.14
		Acid (75%)	83	1.46	6.2	1.46	6.53	0.18
		Hydroprime (24 h)	87	1.44	6.37	0.72	7.57	0.12
		Hydroprime (48 h)	88	1.60	5.10	0.61	7.56	0.14
		Flame + Hydroprime (24 h)	92	1.57	5.02	1.06	6.26	0.23
		Flame + Hydroprime (48 h)	87	1.41	7.26	1.30	6.97	0.10
		Acid (75%) + Hydroprime (24 h)	82	1.54	5.32	1.35	6.30	0.25
		Acid (75%) + Hydroprime (48 h)	81	1.55	4.98	1.18	6.21	0.27
Rytidosperma caespitosum	2	Control	41	2.86	3.01	0.96	6.80	0.55
		Flame (intermittent)	29	5.65	2.11	1.08	8.37	1.27
		Flame (continuous)	34	2.97	3.43	1.34	7.86	0.64
		Acid (50%)	22	2.28	3.64	2.04	6.22	0.82
		Acid (75%)	39	2.27	3.21	1.25	4.97	0.46
	3	Control	39	2.48	4.42	1.48	8.60	0.50
		Flame (continuous)	30	2.83	3.97	1.74	8.88	0.70
		Acid (75%) 2	34	3.25	3.25	1.40	8.20	0.67
		Hydroprime (48 h)	39	2.21	4.55	1.81	7.25	0.44
		Flame + Hydroprime	33	4.91	2.23	1.22	7.19	0.91
		Acid + Hydroprime	38	1.83	4.96	2.95	5.41	0.88

Aristida inaequiglumis		Control	93	1.47	4.51	1.03	2.11	0.05
		Acid 75%	92	1.28	12.71	32.29	1.92	0.21
		Acid 80%	97	1.34	10.83	97.56	1.92	0.75
		Acid 90%	93	1.49	4.32	1.10	2.03	0.04
		Acid 100%	62	1.78	2.81	0.73	2.02	0.10
Chrysopogon fallax		Control	24	2.39	2.04	0.90	3.53	0.56
		Acid 75%	79	3.17	2.57	0.60	6.55	0.28
		Acid 100%	36	1.83	3.67	1.15	4.29	0.27
Cymbopogon ambiguus		Control	97	2.13	3.16	0.54	3.78	0.13
		Flame	100	1.87	5.12	2.35	3.51	0.22
		Acid 50%	72	3.35	1.84	0.35	4.27	0.27
		Acid 75%	91	1.99	2.86	0.39	2.96	0.12
Cymbopogon obtectus	2	Control	93	1.68	3.50	0.49	3.94	0.10
		Flame	96	1.60	4.25	1.10	3.54	0.13
		Acid 50%	93	1.51	3.46	0.63	3.12	0.15
		Acid 75%	95	1.43	3.65	0.59	2.99	0.14
Eriachne obtusa		Control	79	1.96	3.28	0.86	3.4	0.20
		Flame	71	2.05	2.99	0.71	3.57	0.19
		Acid 50%	76	2.02	2.65	0.45	3.25	0.16
		Acid 75%	66	1.61	4.10	1.46	2.95	0.31
Eulalia aurea		Control	87	2.08	3.24	0.34	4.67	0.13
		Flame (low)	88	1.80	4.01	0.61	4.14	0.09
		Flame (high)	83	2.08	3.14	0.51	3.94	0.12
		Acid 50%	77	1.90	3.18	0.75	3.33	0.18
		Acid 75%	81	1.31	4.72	0.82	2.70	0.16

Supplementary Material III. Cost of SET application

Exposure duration for flaming was generally longer that for acid digestion at optimal concentration. However, flaming requires minimal equipment preparation or post-treatment processing (i.e. immediately ready for use following treatment). By contrast, acid digestion requires substantial preparation (e.g. sulphuric acid dilutions and cooling time) and post-treatment processing (e.g. neutralising, rinsing, and drying treated seeds for up to 48 h). Also note that 1 L is the minimum volume of seed material that can be treated at once for flash flaming, while 1 L is the maximum volume that can currently be treated at once for acid digestion using standard laboratory glassware (i.e. max volume 2 L).

Table S6. Breakdown of cost estimates to apply flash flaming and acid digestion to a 1 L volume of seed material as per the methodology used in this research-scale study. All price estimates are provided in AUD.

SET	Estimated time to treat 1 L volume	Post-treatment processing	Materials cost	Estimated cost of materials to treat 1 L volume	Technician cost ($40/h) to treat 1 L volume	Total estimated cost per L
Flash Flaming	10 min	Ready for use	Tradeflame TF/ULTRA GAS Performance Propane Gas - MAPP® = $15/400 g bottle.<br><br>Consumption of up to 295 g/h with small flaming torch [1]. Estimated to give 1-2 hrs of run time = ~$7.5-15/h	1L can be treated in 10 min.<br><br>(10/60)*7.5 to (10/60)*15 = $1.25-2.5/L	Treatment complete in 10 min.<br><br>(10/60)*40 = $6.67	$7.92 - $9.17
Acid digestion	2-10 min	Neutralising, rinsing seeds (~5 min).<br><br>Drying seeds (up to 48 h)	Sulfuric acid SIGMA Chemicals, Perth = $25/L	1 L volume of seed requires 1 L volume of acid solution. Estimate for concentration range of 50% - 100% solution = $12.5-25/L	Estimated time including treatment, neutralising, rinsing and preparing for drying = 10-20 min<br><br>(10/60)*40 to (20/60)*40 = $6.67-$13.33	$19.17 - $38.33

Supplementary Material IV. Seed collection, floret fill, storage and processing

Two separate seed batches of *A. inaequiglumis* were used for different components of the germination experiments due to the small quantities available. Batch 1 (“B1”) was utilised for germination biology tests and pilot studies to determine suitable concentrations and exposure durations for acid digestion (no germination testing) (Table S7). Batch 2 (“B2”) was utilised for germination tests of acid digestion (Table S7). All seed was stored at 15°C and 15% relative humidity until experimental use, though time between collection and arrival at storage facilities is unknown.

Table S7. List of study species (scientific and common name), photosynthetic pathway (C3 or C4), climatic conditions (MAP and MAT) associated with their distribution, collection information (location and collector/supplier), and floret fill as determined by x-ray analysis. Germination testing dates and seed age (y = years, m = months) for each test are provided, with annotations “Ex1”, “Ex2”, and “Ex3” indicating Experiment 1 (germination biology), Experiment 2 (SETs to improve seed handling), and Experiment 3 (SETs to provide additional germination benefits), respectively.

Species name	Common name	C3/C4	Climate (MAP, MAT)	Collection Information	Testing dates	Ages at testing	Floret fill
***Neurachne alopecuroidea* R.Br.**	Foxtail Mulga Grass	C3	250-1300 mm, 13-21°C	South Stirlings, Yarrabee. November, 2019 (collector: Formosa Flora).	August 2020 (Ex1), October 2020 (Ex2), November 2020 (Ex3)	9 m (Ex1), 11 m (Ex2), 1 y (Ex3)	87%
***Rytidosperma caespitosum* (Gaudich.) Connor & Edgar**	Common Wallaby Grass	C3	150-1500 mm, 10-22°C	Boxwood Hill, Jerramungup. November, 2016 (collector: Formosa Flora).	August 2020 (Ex1), October 2020 (Ex2), November 2020 (Ex3)	3 y 9 m (Ex1), 3 y 11 m (Ex2), 4 y (Ex3)	87%
***Aristida inaequiglumis* Domin**	Feathertop Threeawn Grass	C4	150-1550 mm, 20-28°C	**Batch 1:** Great Northern Highway/Yandi Access Road, Newman. March, 2009 (collector: Todd Erickson). **Batch 2:** Ophthalmia Dam, Newman. July, 2008 (collector: Todd Erickson).	March 2021 (Ex1, B1), April 2021 (Ex2, B1), July 2021 (Ex2, B2)	12 y (Ex 1, B1), 12 y 1 m (Ex2, B1), 13 y (Ex2, B2)	89% (Batch 1), 94% (Batch 2)

***Chrysopogon fallax* S.T.Blake**	Ribbon Grass	C4	125-2000 mm, 14-29°C	Port Hedland. March, 2015 (provided by: BHP).	March 2021 (Ex1), April 2021 (Ex2)	6 y (Ex1) 6 y 1 m (Ex2)	41%
***Cymbopogon ambiguus* A.Camus**	Lemon Grass	C4	125-2000 mm, 15-29°C	Marillana. February/March, 2016 (provided by: BHP).	March 2021 (Ex1), April 2021 (Ex2)	6 y 1 m (Ex1), 6 y 2 m (Ex2)	78%
***Cymbopogon obtectus* S.T.Blake**	Silkyheads Lemon Grass	C4	150-1500 mm, 15-28°C	Marillana. June, 2015 (provided by: BHP).	March 2021 (Ex1), April 2021 (Ex2)	5 y 9 m (Ex1), 5 y 10 m (Ex2)	70%
**Eriachne obtusa *R.Br.***	Wire Grass	C4	200-1700 mm, 18-29°C	Port Hedland. March, 2013 (provided by: BHP).	March 2021 (Ex1), April 2021 (Ex2)	8 y (Ex1), 8 y 1 m (Ex2)	75%
***Eulalia aurea* (Bory) Kunth**	Silky Browntop	C4	150-1550 mm, 15-28°C	Juna Downs Station. April, 2018 (provided by: Rio Tinto).	March 2021 (Ex1), April 2021 (Ex2)	2 y 11 m (Ex1), 3 y (Ex2)	55%

Supplementary Material V. Selection of SETs and application methods

Selection of SETs

The germination biology studies highlighted that seed handling (rather than germination) was the most significant challenge to overcome to improve the restoration and commercial success of the study species. Therefore, flash flaming and acid digestion were selected as two suitable treatments known to improve seed handling. Both treatments were tested in species with finer hairs and awns (*N. alopecuroidea, R. caespitosum, C. ambiguus, C. obtectus. E. obtusa,* and *E. aurea*), while species with thickened awns an appendages (*A. inaequiglumis* and *C. fallax*) received acid digestion treatments only (as flaming is ineffective at removing such structures [2]). Flaming and/or acid digestion were also considered suitable for weakening floret structures in *C. fallax* and *E. obtusa* as these species benefited from manual removal of floret structures.

Since neither removal of floret structures or KAR_1 helped to improve maximum germination in *R. caespitosum* or increase germination rate and synchronicity in *N. alopecuroidea*, hydropriming was considered. Germination (maximum and germination rate) for the warm-climate species was generally high or able to be improved via SETs used for seed handling (e.g. acid digestion improved germination in *C. fallax* and *E. obtusa*). Therefore, hydropriming was not explored in the warm-climate species.

Application method selection – flaming

Selection of flame size, exposure duration, and the range of temperatures was based on findings from previous studies [2], and preliminary testing. During preliminary testing, the desired morphological change for all study species had occurred at approx. 10 min at 110 ± 10°C, except in the instance of *E. aurea* where increasing flame temperature assisted in achieving the desired morphological changes within the 10 min exposure duration (Fig. S1). Intermittent flaming was tested for the cool-climate species due to known sensitivity to flaming [2, 3]. The cooling periods during intermittent flaming were achieved by extinguishing the flame whilst the floret material remained in motion inside the flaming machine.

Application method selection – acid digestion

Acid digestion concentrations and exposure durations were selected based on pilot studies performed to evaluate the efficacy of appendage removal. During these studies, concentrations of 50%, 75%, and 100% were prepared (as well as 80% and 90% for *A. inaequiglumis*) by dilution of pure sulphuric acid in reverse osmosis water. Diluting sulphuric acid in water creates a highly exothermic chemical reaction and therefore all dilutions were allowed to cool to room temperature before performing acid digestion treatments (for both pilot studies and final experiments).

Not all concentrations were tested in all species due to differences in appendage thickness. For instance, concentrations >75% were too corrosive for species with fine-haired floret structures, while concentrations <75% were ineffective at removing thickened structures (Fig. S1). Treatments were long enough to ensure sufficient reduction (>80%) or removal of hairs, bristles, awns or appendages, but not so long as to result in complete removal of the palea and lemma (Fig. S1). This was determined based on visual assessment throughout the treatment and the time taken to achieve this change was recorded. Further visual assessment was made once treated florets were thoroughly dry to assist in the final treatment selections for germination testing.

Application method selection – hydropriming

The hydropriming durations selected in this study were largely guided by previous studies showing 48 h to be an effective hydropriming duration in *R. caespitosum* [3]. In these studies 24 h hydropriming was also tested and produced positive results. Therefore, 24 and 48 h were selected for trial in *N. alopecuroidea* in this study, while 48 h only was used for *R. caespitosum*.

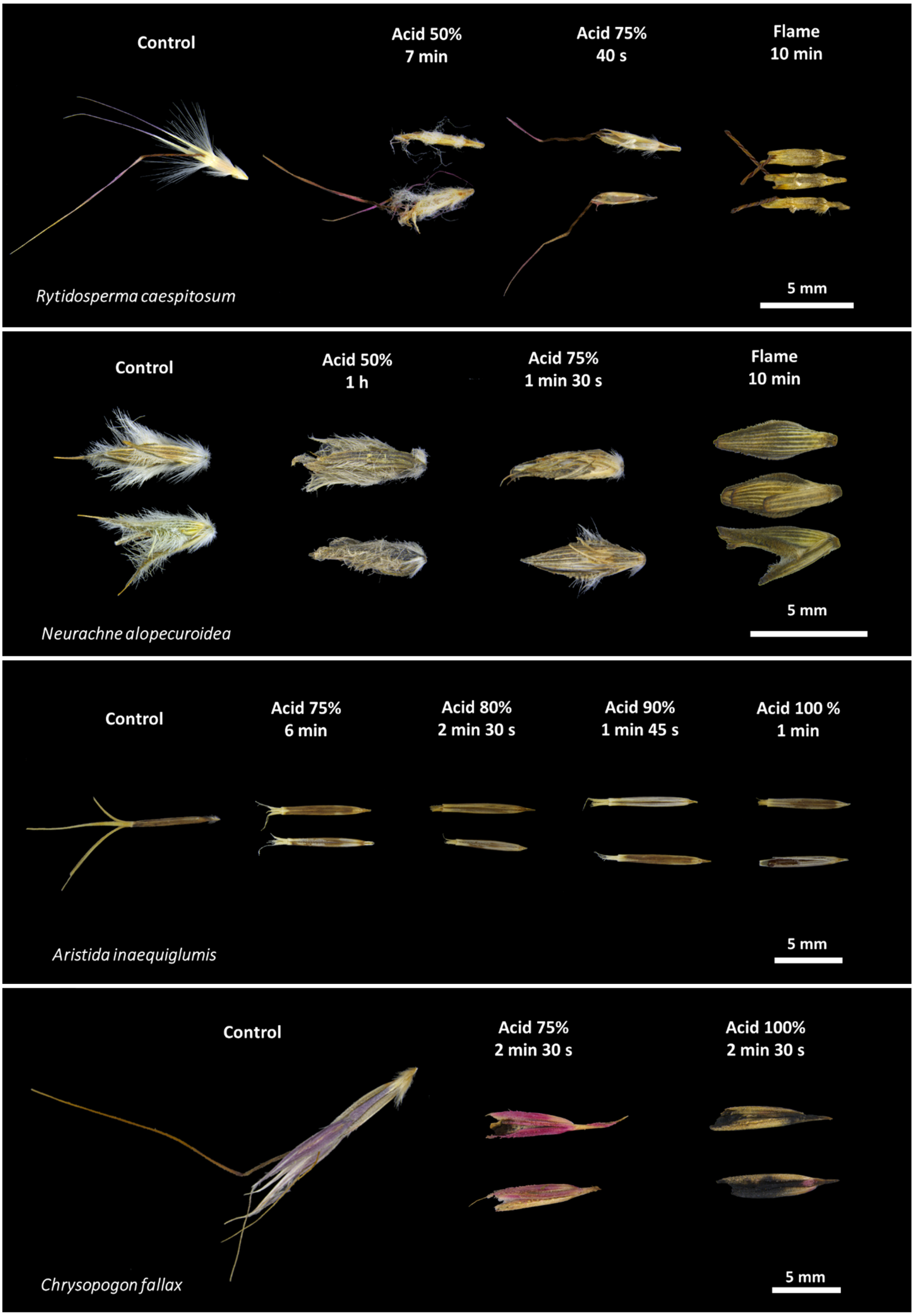

Figure S1. High resolution floret images highlighting the characteristics (awns, hairs, appendages) of each of the study species, and the morphological changes following the different acid digestion and flaming treatments. Figure continued on next page.

Figure S1 continued…

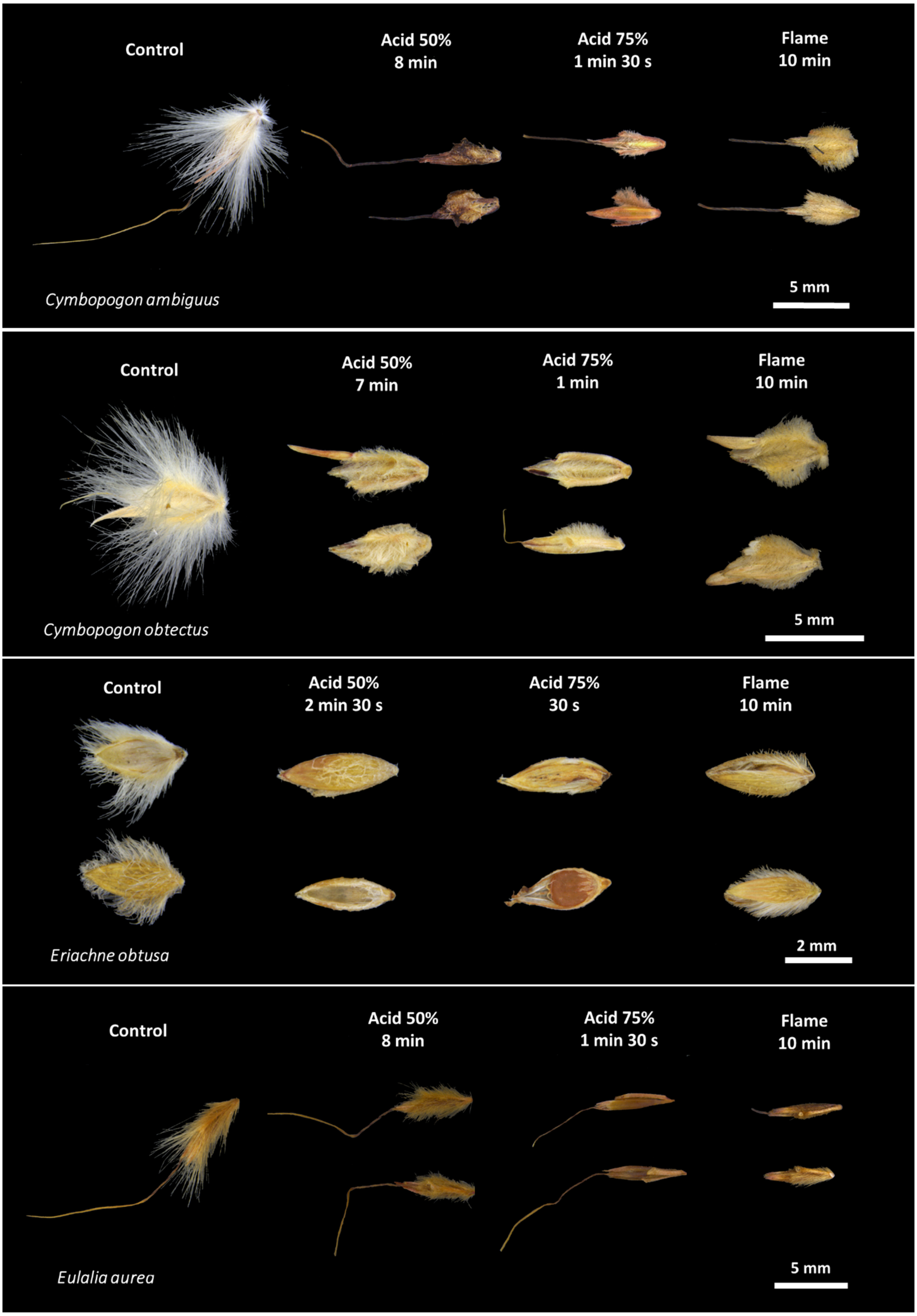

Supplementary Material VI. Flash flaming equipment and techniques

The flash flaming machine consists of a large 900-mm diameter drum with a rotational plate at the base which causes seed material to spin around the drum in a stream via centripetal force (Fig. S2). A plate rotation speed of ~100 rpm was used for all species. The seed stream passes rapidly via the flame to gradually singe off unwanted appendages without exposing seeds to potentially lethal temperatures. For all flaming treatments in this study, a single flaming torch was used and positioned approximately 5 cm away from the stream of seeds. The flame temperature was adjusted simply by increasing or decreasing the flow of gas. Flame temperature was monitored at regular intervals (approx. every 2 min) to ensure consistency throughout and between treatments.

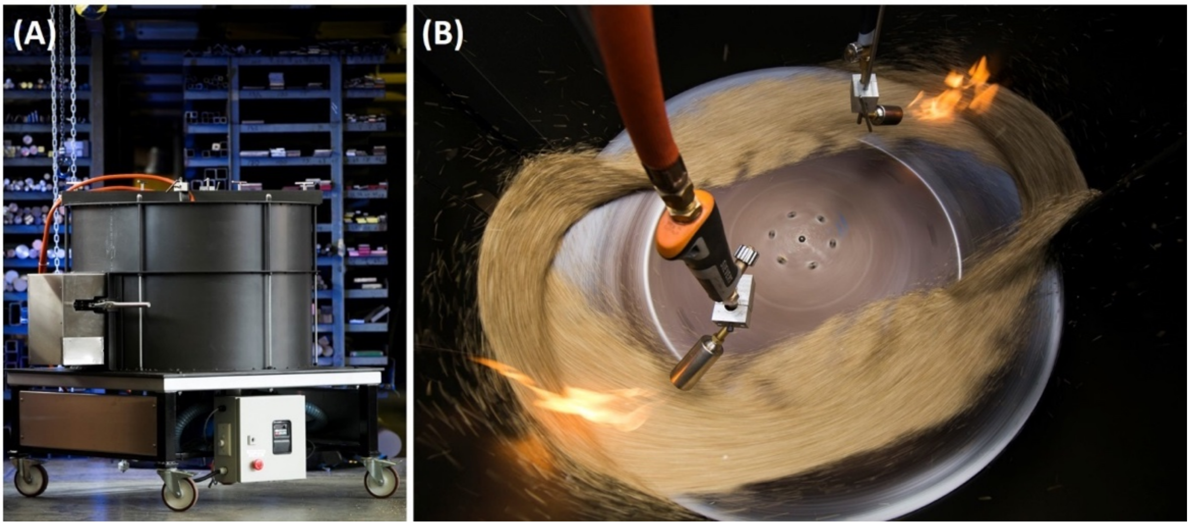

Figure S2. The flaming apparatus, custom-built and supplied by The University of Western Australia, is an up-scaled (900-mm diameter) version (A) of the rotary drum coater described by Guzzomi [4] and Ling [1] with two flaming torches (B). Flame intensity, the angle and distance of the torch relative to the path of florets, and the speed of the rotary plate can all be adjusted. Figure from [5].

Table S8. Volume and weight changes following flash flaming treatments.

Species (treatment where applicable)	Start volume (L)	End volume (L)	Start weight (g)	End weight (g)
Neurachne alopecuroidea (continuous)	1	0.45	37	29
Neurachne alopecuroidea (intermittent)	1	0.40	35	29
Rytidosperma caespitosum (continuous)	1	0.40	26	19
Rytidosperma caespitosum (intermittent)	1	0.30	23	17
Cymbopogon ambiguus	1	0.20	17	10
Cymbopogon obtectus	1	0.15	14	11
Eriachne obtusa	1	0.35	57	33
Eulalia aurea (110 ± 10°C)	1	0.40	Missing data	Missing data
Eulalia aurea (150 ± 10°C)	1	0.20	Missing data	Missing data

Supplementary Material VII. Priming unit

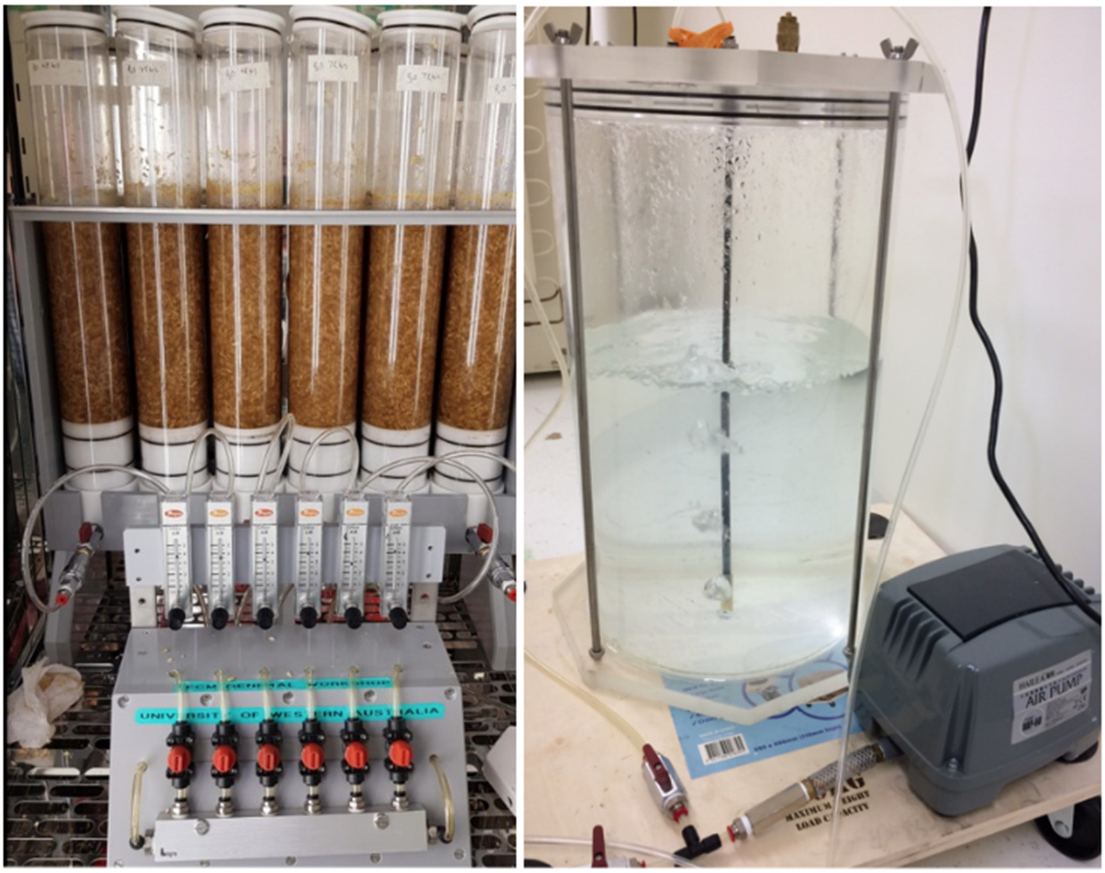

Figure S3. The priming apparatus was custom-built by The University of Western Australia and has six cylindrical tubes, each 3 L in volume (left), and an aeration system capable of delivering continuous humid airflow during priming treatments. Humid air is required during priming to minimise any changes in water potential of the priming solution. To achieve this, air is produced by an aquarium air pump, then pumped via plastic tubing into the lower half of a large cylinder (~ 100 L) filled with approx. 50 L of water (right). The air remaining in the large cylinder is humid and this humid air is used to supply the priming tubes. The airflow to each of the six cylinders can be controlled manually, with a maximum delivery of 5 L/min. Figure adapted from [6].

REFERENCES

1. Ling, E.; A.L. Guzzomi; D.J. Merritt; M. Renton; T.E. Erickson. Flash flaming technology shows promise to improve seed-based rehabilitation outcomes. In Proceedings of the 13th International Conference on Mine Closure. 2019; A.B. Fourie and M. Tibbett; Australian Centre for Geomechanics: Perth.
2. Berto, B.; T.E. Erickson; A.L. Ritchie. Flash Flaming Improves Flow Properties of Mediterranean Grasses Used for Direct Seeding. *Plants*, **2020**. *9*, pp. 1699.
3. Berto, B.; A.L. Ritchie; T. Erickson. Seed-enhancement combinations improve germination and handling in two dominant native grass species. *Restoration Ecology*, **2021**. *29*, pp. e13275.
4. Guzzomi, A.L.; T.E. Erickson; K.Y. Ling; K.W. Dixon; D.J. Merritt. Flash flaming effectively removes appendages and improves the seed coating potential of grass florets. *Restoration Ecology*, **2016**. *24*, pp. S98-S105.
5. Erickson, T.E.; M. Munoz-Rojas; A.L. Guzzomi; M. Masarei; E. Ling; A.M. Bateman; O.A. Kildisheva; A.L. Ritchie; S.R. Turner; B. Parsons; P. Chester; T. Webster; S. Wishart; J.J. James; M.D. Madsen; S.R. Abella; D.J. Merritt. A case study of seed-use technology development for Pilbara mine site rehabilitation. In Proceedings of the 13th International Conference on Mine Closure. 2019; A.B. Fourie and M. Tibbett; Australian Centre for Geomechanics: Perth, Australia.
6. Erickson, T.; O. Kildisheva; O. Baughman; M. Breed; L. Ruiz-Talonia; V. Brown; M. Madsen; D. Merritt; A. Ritchie, *Florabank Guidelines Module 12 - Seed Enhancement Technologies*, in *Florabank Guidelines – best practice guidelines for native seed collection and use (2nd edn)*, L.E. Commander, Editor. 2021, Florabank Consortium: Australia.
